# Absolute Binding Free Energies with OneOPES

**DOI:** 10.1021/acs.jpclett.4c02352

**Published:** 2024-09-20

**Authors:** Maurice Karrenbrock, Alberto Borsatto, Valerio Rizzi, Dominykas Lukauskis, Simone Aureli, Francesco Luigi Gervasio

**Affiliations:** † School of Pharmaceutical Sciences, University of Geneva, Rue Michel-Servet 1, CH-1206 Geneva, CH; ‡ Institute of Pharmaceutical Sciences of Western Switzerland, University of Geneva, CH-1206 Geneva, CH; ¶ Swiss Bioinformatics Institute, University of Geneva, CH-1206 Geneva, CH; § Chemistry Department, University College London (UCL), WC1E 6BT London, U.K.

## Abstract

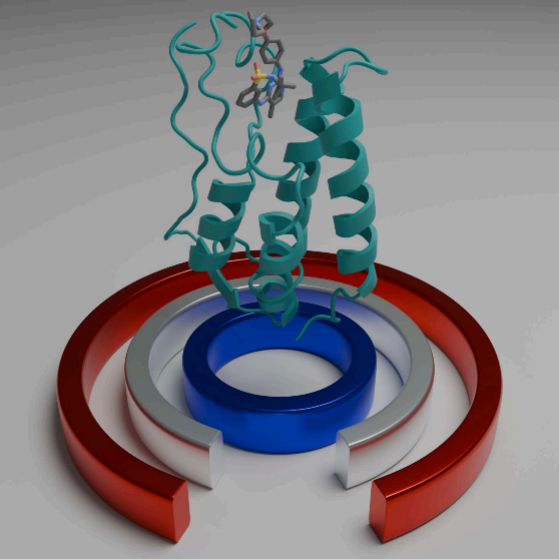

The calculation of absolute binding free energies (ABFEs) for protein–ligand systems has long been a challenge. Recently, refined force fields and algorithms have improved the quality of the ABFE calculations. However, achieving the level of accuracy required to inform drug discovery efforts remains difficult. Here, we present a transferable enhanced sampling strategy to accurately calculate absolute binding free energies using OneOPES with simple geometric collective variables. We tested the strategy on two protein targets, BRD4 and Hsp90, complexed with a total of 17 chemically diverse ligands, including both molecular fragments and drug-like molecules. Our results show that OneOPES accurately predicts protein–ligand binding affinities with a mean unsigned error within 1 kcal mol^–1^ of experimentally determined free energies, without the need to tailor the collective variables to each system. Furthermore, our strategy effectively samples different ligand binding modes and consistently matches the experimentally determined structures regardless of the initial protein–ligand configuration. Our results suggest that the proposed OneOPES strategy can be used to inform lead optimization campaigns in drug discovery and to study protein–ligand binding and unbinding mechanisms.

The binding and unbinding of ligands to protein targets underlie both biological and pharmaceutical activity. Accurate prediction of protein–ligand binding free energies is therefore a critical aspect of computer-aided drug design (CADD). For instance, the calculated free energies can be used to prioritize compound synthesis and to guide hit optimization.^1−4^ Recent improvements in force fields, sampling algorithms, and the advent of low-cost parallel computing have improved the quality of simulation-based free energy predictions and made them more affordable. However, further advances are still needed to achieve reliable and accurate results for a wide range of ligands and protein targets.^5^

Despite the availability of high-quality protein force fields, major challenges remain in the parametrization of complex ligands, including those with delocalized electron density.^5,6^ In addition, the interaction between suboptimal ligand, protein, and solvent force fields can introduce artifacts and systematic errors that are difficult to account for. With respect to free energy calculations, unbiased molecular dynamics (MD) would require impractically long simulation times to exhaustively sample all the relevant states contributing to the free energy of binding before providing reliable estimates.^7−10^ To address this issue, several MD-based methods have been developed, such as alchemical transformations^11−16^ and collective variable-based enhanced sampling approaches.^17−24^

The former decouple the ligand from the protein along a nonphysical thermodynamic cycle, known as the alchemical cycle. This allows the calculation of binding free energies without directly simulating the binding and unbinding events. This eliminates the need to sample the intermediate states that are often found along physical uncoupling pathways, leading in many cases to accurate free energy estimates at a reasonable computational cost.^14^ However, these methods face problems when the ligand binding pose is not known or there are significant conformational changes in the target following the formation/disruption of the protein–ligand complex.^25^

Collective variable-based enhanced sampling approaches, on the other hand, directly accelerate the binding and unbinding of the ligand to the target protein along physical association pathways. These strategies rely on the definition of a set of collective variables (CVs) that approximate the reaction coordinate and drive exploration of the underlying free energy surface, effectively accelerating the transitions between different metastable states. In principle, they do not depend on the knowledge of the correct binding pose, but their performance is highly dependent on the quality of the CVs and is typically limited to the simultaneous use of no more than three CVs.^26^ In the case of ligand binding, typical CVs include the distance of the ligand from the pocket as well as its orientation and conformation. Other important factors that have been shown to play a role in many binding mechanisms are the ligand and cavity solvation as well as the conformation of the cavity.^27−31^

If the selected CVs fail to accelerate the sampling of the relevant slow (and often unknown) degrees of freedom, then convergence of the free energy landscapes associated with binding and unbinding of the ligand may require impractically long sampling times. In the most unfavorable cases, no convergence is observed.^32−34^ Defining an optimal set of CVs is challenging, especially since the optimal set might vary for different ligands binding to the same target. Other aspects that complicate the use of CV-based algorithms include monitoring the convergence of the estimated free energy profiles and determining when to stop the calculation.^35^ In addition, regardless of the approach, the presence of multiple competing binding poses, interfacial water, or large conformational changes in the protein and in the ligand tend to affect the reliability of the free energy estimates.^9,10,28,36−40^

To address the challenge of defining optimal yet universally applicable CVs for ligand binding as well as other issues that hinder the use of CV-based approaches for absolute binding free energy calculations, we present a transferable enhanced sampling strategy. Our approach leverages our recently developed OneOPES (One On-the-fly Probability Enhanced Sampling) algorithm^41^ and a simple set of geometry-based CVs to calculate the absolute binding free energies of protein–ligand complexes. Specifically, OneOPES combines multiple replicas with different versions of OPES^42−44^ to reduce the dependence of the free energy estimates on an optimal choice of CVs, while the designed set of simple geometric CVs can be easily adapted to most ligand-target systems.

Here, we test the approach on a set of 17 protein–ligand complexes comprising two well-characterized protein systems: bromodomain-containing protein 4 (BRD4) and heat shock protein 90 (Hsp90). The set includes 11 BRD4-ligand complexes and 6 Hsp9-ligand complexes, including both experimentally determined and modeled binding poses, with ligands varying widely in size and affinity for the target proteins. This diversity allows for a thorough evaluation of our computational strategy. Additionally, the test set includes two different crystallographic poses of the same ligand bound to Hsp90 (PDB IDs: 2WI2 and 2WI3). This system allows for a direct assessment of the accuracy of our method across different initial ligand orientations within the binding pocket. We also tested two different force fields and two water models to assess their impact on the resulting free energy estimates.

Our results correlate well with experimental data, with a mean unsigned error within 1 kcal mol^–1^ of experimentally determined free energies. The results highlight how our OneOPES protocol provides accurate absolute binding free energy estimates in agreement with experimental values across a wide range of systems. Our strategy can efficiently accelerate the sampling of a number of relevant degrees of freedom without the need for the time-consuming development of *ad hoc* CVs. Ultimately, the transferability of our protocol suggests a promising route toward its application to lead optimization campaigns in drug discovery.

**OneOPES for Ligand Binding.** OneOPES was originally designed to address the problem of converging free energy landscapes with a suboptimal set of CVs.^41^ It combines a CV-driven exploration with a replica exchange strategy and tempering scheme to accelerate the crossing of significant free energy barriers. Here, we adapted the original OneOPES approach to accelerate the binding and unbinding of ligands to a target protein without having to tailor the set of CVs for each ligand-target system. Specifically, we designed two general CVs that describe the orientation and distance of the ligand relative to the target protein pocket (Figure 1). These CVs are used in OneOPES to control the main simulation bias that is deposited via the OPES Explore^44^ sampling scheme.

**Figure 1:**
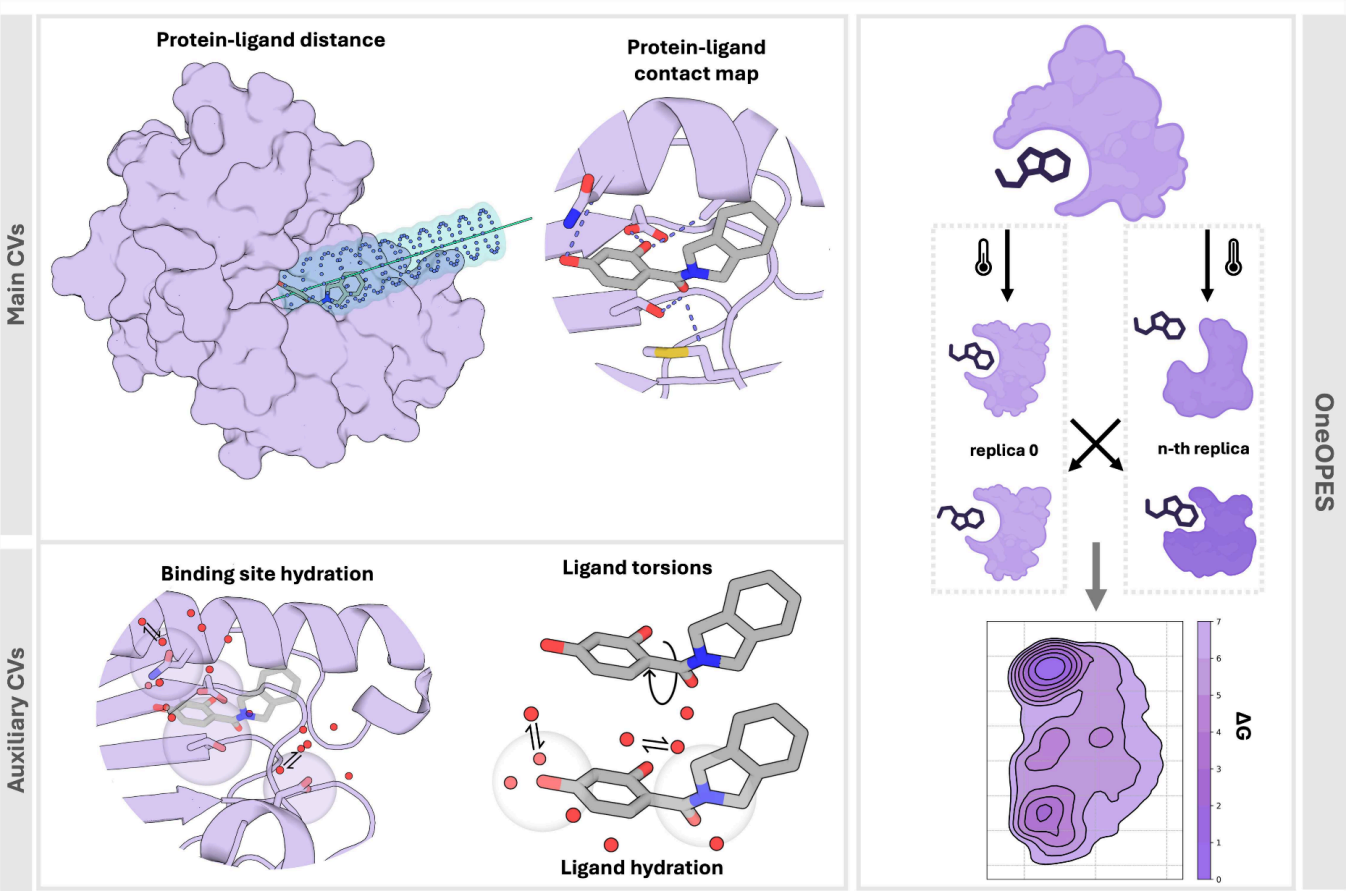
**Schematic of our OneOPES strategy for protein–ligand binding.***Top-left panel:* example of the two main CVs biased with OneOPES, the protein–ligand distance, shown as a cyan line, and the protein–ligand contact map. A funnel-shaped restraint is applied along the protein–ligand distance vector. *Bottom-left panel:* examples of the auxiliary CVs accelerated with OneOPES, i.e., hydration sites, both within the protein binding site and some ligand atoms, and ligand torsions. *Right panel:* illustration of the replica exchange and the thermal gradient used in our OneOPES simulations. The combination of different enhanced sampling schemes allows for the exploration of several ligand binding modes, resulting in accurate binding free energy surfaces.

The first CV describes the distance between the center of mass of the ligand and the binding site. The center of mass of the ligand is projected on a vector originating from the protein and intersecting the binding site. The distance between the ligand’s center of mass and the origin of this vector defines the CV and allows us to distinguish between conformations where the ligand is bound to the protein or sampling bulk water. Additionally, this vector serves as the main axis for a funnel-shaped potential, which limits the ligand exploration to relevant regions of the conformational space.^45−47^

The second CV is a contact map (CMAP) that captures the initial orientation of the ligand within the binding pocket. The contact map is defined such that only ligand orientations similar to the initial binding pose have large CMAP values with the CV decreasing sharply to zero for dissimilar orientations. This property of the CMAP allows reasonable resolution of different ligand poses within the binding pocket as well as the evaluation of poses that differ from the initial one.

In addition to the two primary CVs, our strategy includes additional CVs to improve the sampling of various degrees of freedom that may contribute to the binding and unbinding mechanisms. In particular, we accelerate the water coordination around polar atoms within the binding site, the hydration of polar atoms of the ligand, and the torsion angles that control the transitions between different ligand rotamers. The sampling of different solvation states of the protein cavity is often a crucial aspect of binding and unbinding kinetics.^27−29,31,48^ Water molecules may be essential in bridging favorable interactions between protein side chains and the ligand, thereby stabilizing the bound state. On the contrary, the presence of highly ordered water molecules in the apo binding cavity may constitute a major barrier to ligand rebinding.^48^

Transitions between different ligand conformations, especially in the case of larger ligands, can significantly contribute to the binding free energy.^49^ Typically, specific rotamers correspond to the ligand-bound conformation, with higher affinity ligands usually adopting a single primary rotameric conformation and lower affinity binders exploring a few. However, good quality sampling requires a ligand to explore different rotameric conformations, especially upon unbinding and transitioning to the bulk solution. To this end, we explicitly bias the ligands’ rotatable bonds that are part of delocalized double bonds and are typically associated with higher energy barriers. These auxiliary CVs help to sample different unbound ligand conformations, thereby improving the accuracy of the resulting free energy estimates.

The bias deposited along the auxiliary CVs is significantly lower than the bias deposited along the primary CVs. Similarly, the rate of bias deposition along the additional CVs is lower than the rate of deposition along the primary CVs. In fact, the role of the auxiliary CVs is to accelerate important degrees of freedom that are not explicitly captured by CMAP and the protein–ligand distance. Our OneOPES strategy does not aim to converge the free energy along these auxiliary CVs but employs them only to help converge the 2D free energy projected onto the two primary CVs, where most of the simulation bias is deposited.

To further facilitate the convergence of the binding free energy, OneOPES combines the CV-driven exploration of OPES Explore with eight exchanging replicas and OPES MultiThermal.^43^ The replica exchange scheme accelerates sampling via parallel exploration of the accessible phase space. Furthermore, the addition of an OPES MultiThermal bias renders OneOPES in a way analogous to parallel tempering techniques,^50−54^ effectively allowing the exploration of a user-defined temperature range. By enhancing the fluctuations of the system’s potential energy, OPES MultiThermal allows the sampling of a multicanonical ensemble spanning temperatures within a given temperature range [*T*_min_, *T*_max_].

The different replicas follow an increasingly aggressive exploration gradient, with replica zero being driven only by the OPES Explore bias along the two main CVs and higher replicas being subject to gradually increasing out-of-equilibrium conditions. Specifically, replicas one-seven (i.e., the exploration-dedicated replicas), while subject to the same OPES Explore bias as replica zero, also include additional OPES Explore bias deposited along the auxiliary CVs, with higher replicas containing a greater number of auxiliary CVs. In addition, the exploration-dedicated replicas are also subject to the OPES MultiThermal bias, which enhances the sampling of all degrees of freedom by effectively increasing the sampling temperature, with higher replicas being subjected to higher temperatures. This framework allows rapid exploration of the bound and unbound states across the different replicas, thereby increasing the overall sampling quality and facilitating the convergence of the binding free energy. Finally, upon convergence, the equilibrium quantities, such as the free energy of binding, can be recovered from replica zero via a standard reweighting procedure. Further details on system and simulation setup can be found in Supporting Information Sections 1−4. To determine when to stop the simulations and save computational time we have developed an error-informed stopping strategy that is presented in more detail in Supporting Information Section 5.

**BRD4 and the Impact of the Water Model.** Given the crucial role of water in the binding/unbinding mechanism and the importance of a well-balanced set of ligand, protein, and water force fields,^30,36,48,55−61^ we evaluated the effect of two different water models on the binding affinity estimates of 11 BRD4-ligand complexes (Figure 2a). The set of inhibitors considered includes drug-like molecules with a wide range of physicochemical properties. This collection represents various chemical groups, and the heterogeneity of the set ensures that our results are not overly influenced by limited chemical diversity. Additionally, each BRD4 ligand complex has an experimentally measured binding affinity, and for ten of the 11 complexes the binding mode has been crystallographically determined at high resolution (Table 1). This provides an ideal scenario for retrospectively testing the performance of our strategy and investigating the effects of different combinations of water models and force fields.

**Figure 2:**
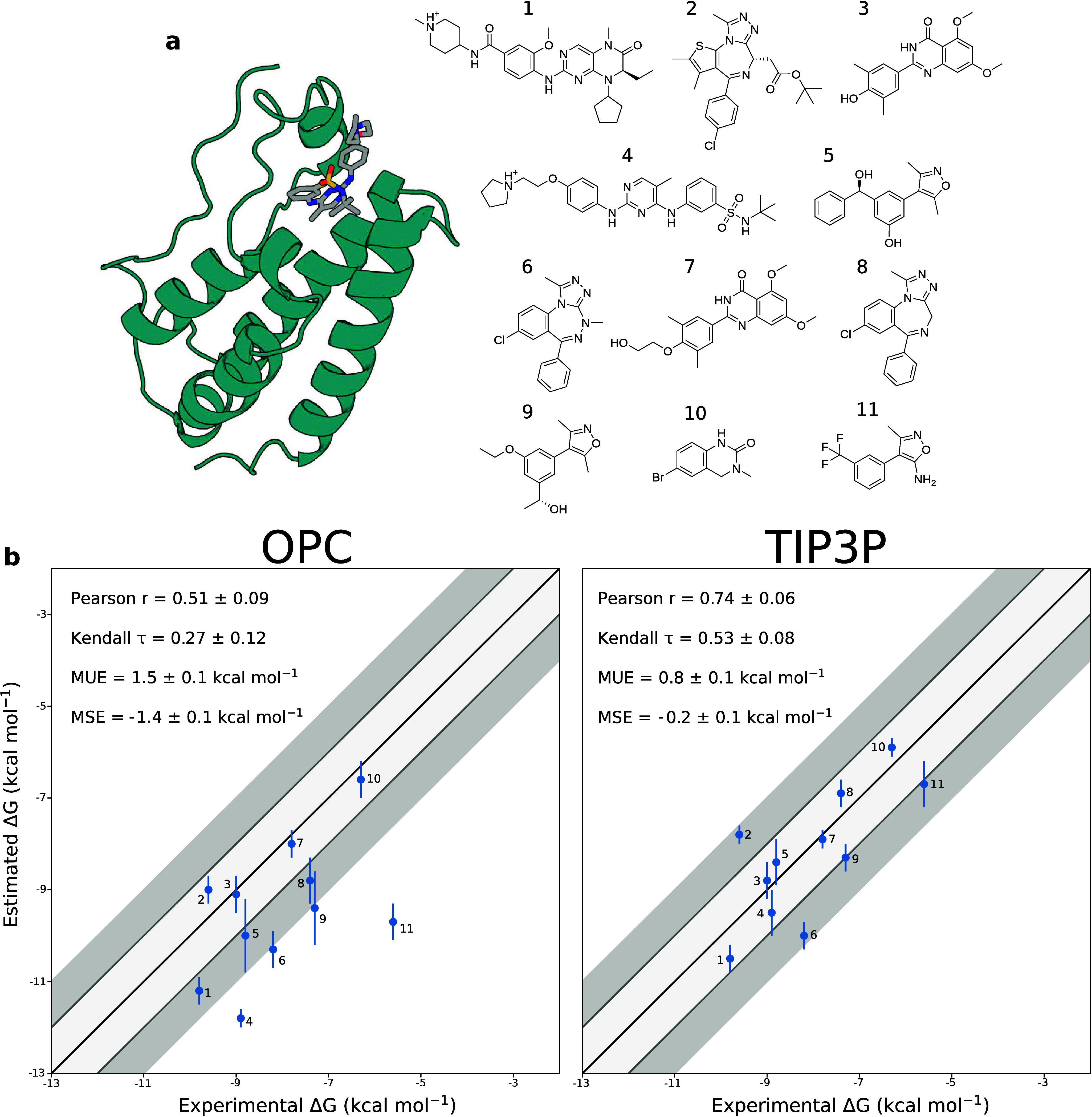
**Structures of the BRD4-ligand complexes and free energy correlation plots.(a)** Structural representation of a BRD4-ligand complex (left, PDB ID: 4OGJ) and chemical structure of the ligands presented in this work (right). The secondary structure of the protein is shown in cyan, and the ligand is shown as sticks. Carbon, nitrogen, oxygen, and sulfur ligand atoms are shown in gray, blue, red, and yellow, respectively. The different ligands are listed in decreasing order of affinity for the BRD4 binding site. **(b)** Correlation plots of experimental versus calculated binding free energies obtained using OneOPES and either the OPC (left) or TIP3P (right) water model. The dark gray shaded area represents a deviation of ±2 kcal mol^–1^ from the experimental values, while the light gray corresponds to a deviation of ±1 kcal mol^–1^. The ideal correlation is shown as a black line.

**Table 1:** **Summary of the BRD4 binding free energy results using OneOPES**^a^

Compound	Δ*G*_*calc*_^*TIP*3*P*^	Δ*G*_*exp*_	Δ*G*_*calc*_^*TIP*3*P*^ – Δ*G*_*exp*_	PDB	μs per replica
1	–10.5 ± 0.3	–9.8 ± 0.1^65^	–0.7	4OGI	0.5
2	–7.8 ± 0.2	–9.6 ± 0.1^66^	1.8	3MXF	0.7
3	–8.8 ± 0.4	–9.0 ± 0.1^67^	0.2	4MR3	0.5
4	–9.5 ± 0.5	–8.9 ± 0.1^65^	–0.6	4OGJ	0.6
5	–8.4 ± 0.5	–8.8 ± 0.1^68^	0.4	4J0R	0.5
6	–10.0 ± 0.3	–8.2 ± 0.1^69^	–1.8	3U5L	1.4
7	–7.9 ± 0.2	–7.8 ± 0.1^67^	–0.1	4MR4	0.6
8	–6.9 ± 0.3	–7.4 ± 0.1^69^	0.5	3U5J	0.5
9	–8.3 ± 0.3	–7.3 ± 0.0^68^	–1.0	3SVG	0.5
10	–5.9 ± 0.2	–6.3 ± 0.1^70^	0.4	4HBV	0.7
11	–6.7 ± 0.5	–5.6^71^	–1.1	Model	0.7

a Δ*G*_*calc*_^*TIP*3*P*^ represents the calculated standard binding free energy with the TIP3P water model; Δ*G*_*exp*_ denotes the experimental standard binding free energy with references provided. The PDB files used as input are listed. Errors in the experimental measurements are reported as one standard deviation, where available. Errors for the calculated standard free energies were obtained using block analysis. All values are given in kcal mol^–1^. The simulation time for each replica (eight exchanging replicas per simulation) is reported.

First, we selected the GAFF2 force field for ligand parametrization and tested it with the latest Amber protein force field, ff19SB, together with the recommended water model, OPC, as this combination is expected to provide higher accuracy. Despite the generally good results, our OneOPES calculations with the OPC water model systematically overestimate the binding free energies, with a mean unsigned error (MUE) of 1.5 ± 0.1 kcal mol^–1^ and a mean signed error (MSE) of −1.4 ± 0.1 kcal mol^–1^ (Figure 2b and Table S1). Additionally, the analysis of the corresponding 2D free energy surfaces revealed that the crystallographic pose of ligand 10 did not correspond to a minimum in the free energy surface (Figure S1).

We then explored the effect of a second water model, namely, TIP3P,^62^ on the free energy calculations, while keeping all other simulation parameters unchanged. The choice of the TIP3P model was further supported by the fact that the GAFF2 parameters were derived with this water model.^63,64^ The resulting binding free energy estimates show a significant improvement over those obtained with the OPC water model, with an MUE of 0.8 ± 0.1 kcal mol^–1^ (Figure 2b and Table 1). Remarkably, the systematic overestimation observed with the OPC water drops significantly, as indicated by an MSE of −0.2 ± 0.1 kcal mol^–1^. Additionally, the simulations correctly identify the crystallographic pose of ligand 10 as the deepest free energy minimum (Figure S1).

For eight of the 11 systems, the error is within 1 kcal mol^–1^ of the experimental value, with only three systems having errors within 2 kcal mol^–1^ (Table 1). In the case of ligand 6, the observed deviation is probably due to the incorrect free energy minimum assigned by the force field (Figure S2 and Table S2). As previously reported, the crystal-like configuration is less favorable for this ligand, with the incorrect binding mode captured by the force field substantially affecting the free energy estimate.^25^ Here we report the binding free energy obtained by considering only the crystal-like configurations of ligand 6. Nevertheless, the bias deposited along the OneOPES simulation still suffers from the incorrect force field parametrization, which probably explains the observed deviation from the experimental value.

In Supporting Information Section 6, we also show the results of analogous simulations performed with Well-Tempered Metadynamics. We ran three independent simulations of 1 μs each, biasing the same primary CVs and using the same *C*_α_-RMSD and funnel-shaped walls. As shown in Supporting Information (Figure S3), the predicted free energies have a higher MUE of 1.7 kcal mol^–1^. Additionally, the uncertainty values of the resulting free energies, calculated as the standard deviation of three estimates, range between 1.5 and 2.4 kcal mol^–1^ for most protein–ligand complexes (Table S3). While the averages over three independent simulations are not too far from experimental values, individual replicas show high variability in the estimates. The impact of the quality of the chosen CVs on the convergence of single replica Metadynamics is indeed well-known and is one of the main motivations for the development of algorithms that combine multiple replica and CV-based enhanced sampling algorithms.^32,35^

To validate our error-informed stopping strategy, we extended the OneOPES simulations to 2 *μs* per replica and recalculated the corresponding binding free energies. The results obtained from the extended calculations are statistically equivalent and strongly correlated, with a Pearson’s *r* of 0.93 ± 0.01, to those obtained with shorter OneOPES simulations (Figure S4). Overall, our error-informed OneOPES strategy significantly reduces the computational cost associated with obtaining accurate binding free energies. Indeed, all OneOPES simulations, except for ligand 6, reached an uncertainty within 1 kcal mol^–1^ in less than 700 ns per replica.

**HSP90 and the Sampling of Multiple Ligand Binding Modes.** To test the transferability of our strategy to other systems, we applied the same OneOPES protocol to six Hsp90-ligand complexes. The selected inhibitors include diverse fragment-like molecules often found in drug-like compounds, with a range of affinities from high *μM* to low *nM* (Figure 3a and Table 2). Additionally, to investigate the effects of multiple ligand binding poses, we selected a protein–ligand system where the ligand was crystallized in two different binding modes within the same binding cavity of Hsp90 (PDB IDs: 2WI2 and 2WI3). Due to the significantly better results obtained previously with TIP3P, we chose this water model for the Hsp90 simulations and parametrized the protein with the recommended Amber ff14SB force field. Since the ff14SB and ff19SB protein force fields are similar, we do not expect significant differences with respect to results obtained with ff19SB.

**Table 2:** **Summary of the Hsp90 binding free energy results using OneOPES**^a^

Compound	Δ*G*_*calc*_	Δ*G*_*exp*_	Δ*G*_*calc*_ – Δ*G*_*exp*_	PDB	μs per replica
1	–9.5 ± 0.7	–9.9^72^	0.4	3K99	0.7
2	–9.3 ± 0.7	–9.1^73^	–0.2	3OW6	1.75
3	–8.1 ± 0.5	–9.1^74^	1.0	3EKO	0.6
4	–9.5 ± 0.3	–8.1^75^	–1.4	2XHT	0.5
5a	–5.7 ± 0.5	–4.7^76^	–1.0	2WI2	0.5
5b	–5.9 ± 0.4	–4.7^76^	–1.2	2WI3	0.5

a Δ*G*_*calc*_ represents the calculated standard binding free energy; Δ*G*_*exp*_ denotes the experimental standard binding free energy, with references provided. The PDB files used as input are listed. Errors for the calculated standard free energies were obtained using block analysis. All values are given in kcal mol^–1^. The simulation time for each replica (eight exchanging replicas per simulation) is reported.

**Figure 3:**
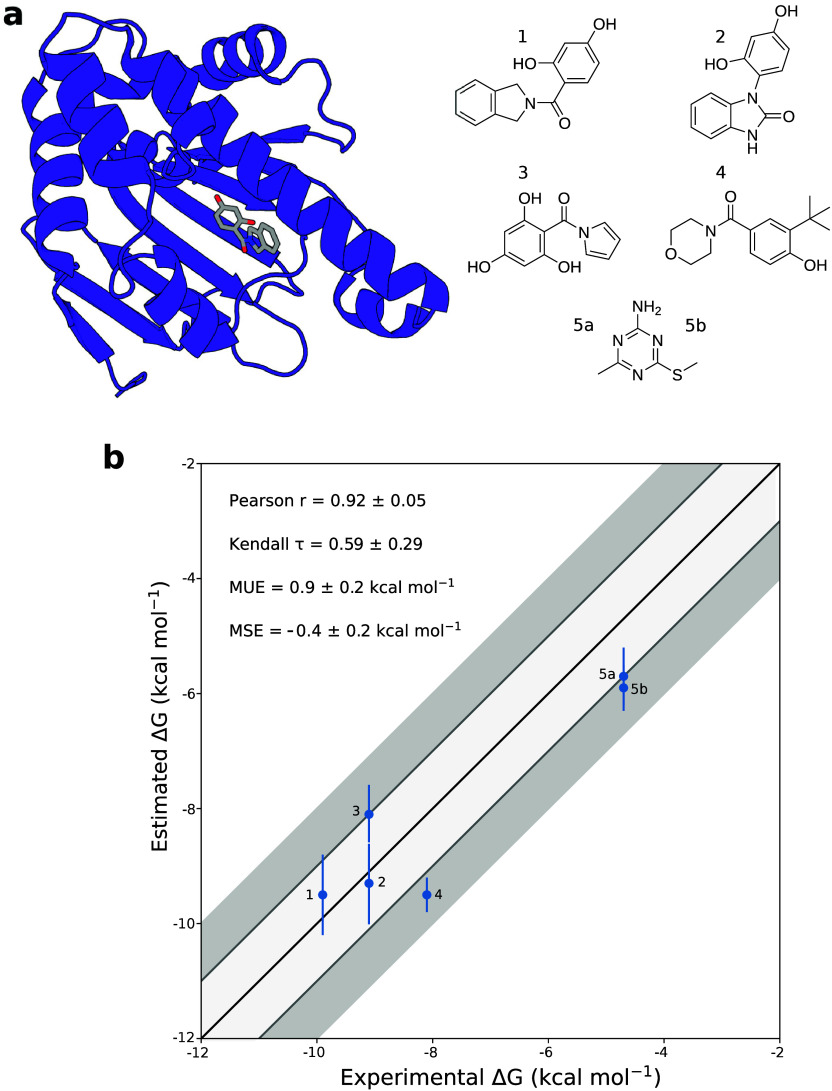
**Structures of the Hsp90-ligand complexes and free energy correlation plots.(a)** Structural representation of a Hsp90-ligand complex (left, PDB ID: 3K99) and chemical structure of the ligands presented in this work (right). The secondary structure of the protein is shown in purple, and the ligand is shown as sticks. Carbon, nitrogen, and oxygen ligand atoms are shown in gray, blue, and red, respectively. The different ligands are listed in descending order of affinity for the Hsp90 binding site. **(b)** Correlation plots of experimental versus calculated binding free energies obtained using OneOPES. The dark gray shaded area represents a deviation of ±2 kcal mol^–1^ from the experimental values, while the light gray corresponds to a deviation of ±1 kcal mol^–1^. The ideal correlation is shown as a black line.

The results show very good agreement with the experimentally determined values (Figure 3b). The calculated mean unsigned error is again within 1 kcal mol^–1^ of the experimental values, in agreement with the previous results obtained from the BRD4 simulations. Specifically, four of the six predictions have errors within 1 kcal mol^–1^ of the experimental measurements, with two having errors within 1.5 kcal mol^–1^ (Table 2). The calculated free energies strongly correlate with the experimental ones, with a Pearson’s *r* of 0.92 ± 0.05, and effectively rank the ligand affinities (Kendall’s τ = 0.59 ± 0.29). The simulation time required to converge the error is also encouraging, with all but one simulation reaching an uncertainty threshold of 1 kcal within 700 ns.

Remarkably, all the experimental starting complexes corresponded to a minimum in the obtained free energy projections, indicating good parametrization of the different systems. Furthermore, complexes 5a and 5b correspond to two different binding modes of the same ligand. Given that the crystal structures capture two different binding poses, we expect that the corresponding binding free energies should be similar, leading to observable populations for both minima.

Indeed, the binding free energies calculated with OneOPES for the two poses are comparable, amounting to 5.7 ± 0.5 and 5.9 ± 0.4 kcal mol^–1^ for poses 5a and 5b, respectively. Interestingly, both free energy estimates converge within the uncertainty of 1 kcal mol^–1^ in the same simulation time (Figure 4a). Moreover, the resulting 1D free energy projections as a function of the protein–ligand distance are superimposable regardless of the starting structure used for the simulations (Figure 4b). This is further supported by the 2D free energy map, where we reprojected the binding free energy as a function of the protein–ligand orientation and the distance from the protein binding site (Figure 4c). Notably, both poses of ligand 5 correspond to a minimum in the free energy map, with longer OneOPES simulations converging to the same free energy landscape independently of the initial binding mode (Figure S5).

**Figure 4:**
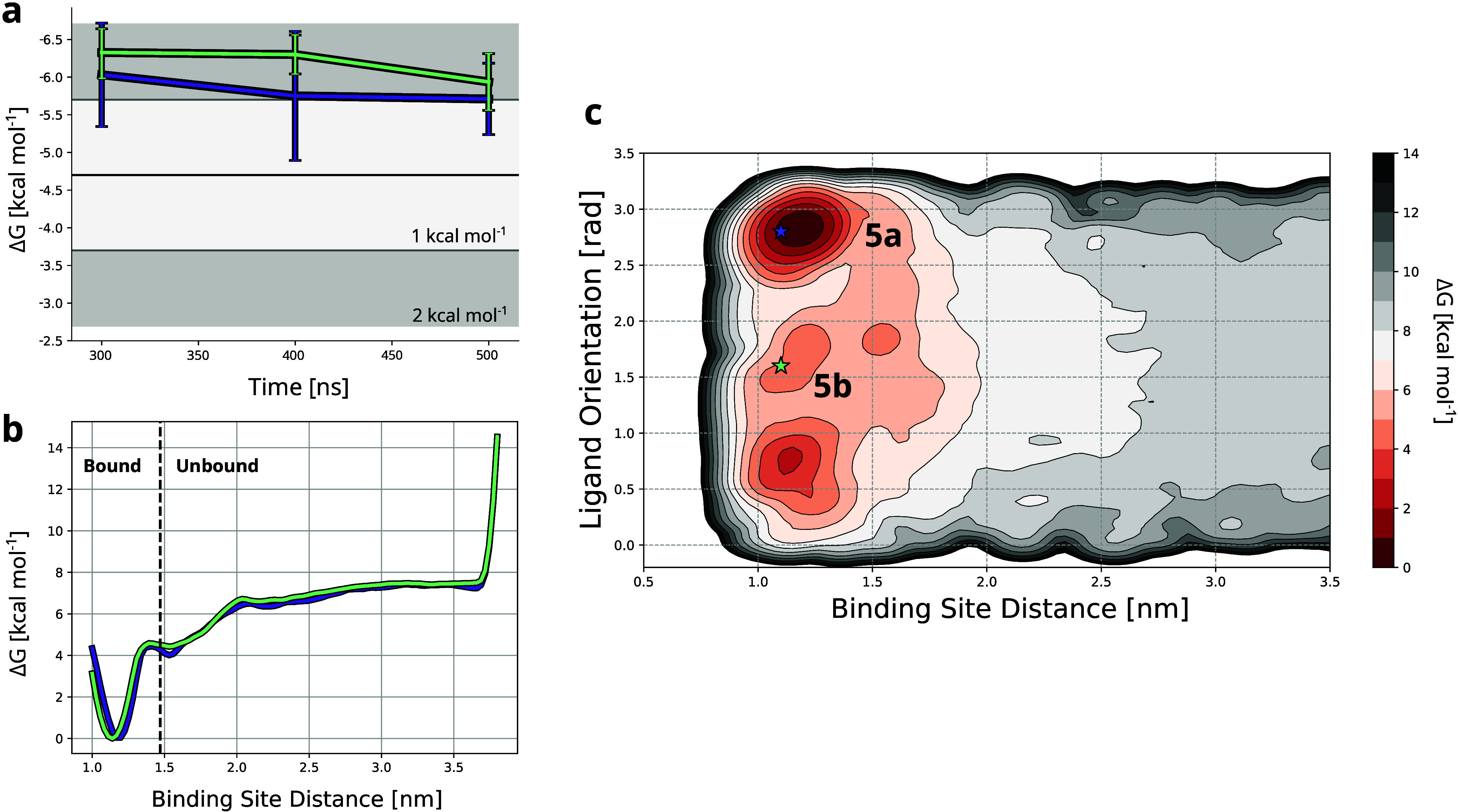
**Sampling of different ligand binding modes with OneOPES.(a)** Binding free energy estimates as a function of different simulation times of the two binding poses of ligand 5. The purple and green lines correspond to two different OneOPES simulations starting from binding poses 5a and 5b, respectively. The first 200 ns of the simulation were considered as equilibration (see Table S5). The dark gray shaded area represents a deviation of ±2 kcal mol^–1^ from the experimental values, while the light gray corresponds to a deviation of ±1 kcal mol^–1^. The experimental binding free energy is shown as a black line. **(b)** 1D free energy projection on the ligand-binding site distance for the two simulations of binding poses 5a and 5b, shown in purple and green, respectively. The distance at which the ligand is considered unbound is indicated by the dashed black line. **(c)** 2D free energy map for the binding of ligand 5 to Hsp90, plotted as a function of the ligand’s distance from the binding site and its orientation. The minima corresponding to the two different binding modes are marked by purple and green stars, representing poses 5a and 5b, respectively. The free energy map was obtained from the simulation starting from binding pose 5a.

Obtaining comparable binding free energies when starting simulations from different initial poses is far from trivial. This highlights how our OneOPES simulations can effectively explore the two crystallographic minima, independently of the starting configuration, and sample different binding modes.

**Conclusions.** CV-based absolute ligand binding calculations have a number of potential advantages over alchemical calculations, such as the ability to explore alternative binding poses and compute the free energy profile along physical association pathways, but their convergence crucially depends on the choice of the set of CVs, which is far from trivial and system dependent. Moreover, monitoring the convergence of the reconstructed free energy landscape is not always straightforward, and consequently it is uncertain when the simulation can be safely terminated.

Here we show that a OneOPES strategy with a set of simple and universal CVs based on distance and contacts performs well for a number of realistic protein–ligand binding systems. Its multireplica nature and the use of auxiliary CVs targeting ligand conformation and hydration further enhance the sampling of complex landscapes that may harbor different local minima, complementing the limitations of the system-agnostic main CVs. We also show that under the same conditions, three independent 1 μs-long single replica Metadynamics calculations give a higher MUE. When combined with an effective strategy to monitor the progress of the free energy estimate and its error, OneOPES produces accurate and reliable free energy results in less than 700 ns of sampling per replica in most cases. The accurate and reliable results have also allowed us to select a combination of ligand and water force fields (TIP3P and GAFF2) that leads to free energy estimates that strongly correlate with the experiments.

The proposed strategy is capable of reconstructing the same (correct) free energy landscape irrespective of the starting pose and properly predicts multiple binding poses when they have been shown to be present by experiments. Thus, OneOPES brings a fresh perspective into the challenge associated with estimating ligand binding free energies, especially in complex systems characterized by nontrivial binding poses and unidentified slow degrees of freedom. A promising future direction is the study of systems with cryptic binding sites.^77^ By studying the complex conformational changes of these pockets alongside ligand binding, it would be possible to gain a more comprehensive understanding of the binding mechanisms. A method that is able to reliably converge the ligand binding free energy profile along physical association pathways can be used as a reference in a number of computational and drug discovery applications, including the training of machine learning approaches.

## Data Availability

The Gromacs and Plumed input files used, and the structures are available on Zenodo at: 10.5281/zenodo.13620043 and on the PLUMED-NEST repository https://www.plumed-nest.org/eggs/24/017/.

## Supporting Information Available

Additional figures, tables, and computational details (PDF)
Transparent Peer Review report available (PDF)
