# Supplementary material for: Absolute Binding Free Energies with OneOPES
Source: J Phys Chem Lett. 2024 Sep 20;15(39):9871–80. doi: 10.1021/acs.jpclett.4c02352 (PMC11457222; doi:10.1021/acs.jpclett.4c02352)
Supplement: Supplementary file 1 — jz4c02352_si_001.pdf [file jz4c02352_si_001.pdf]

Supporting Information: Absolute Binding Free Energies with OneOPES

Maurice Karrenbrock[∇,†,‡,¶] Alberto Borsatto[∇,†,‡,¶] Valerio Rizzi,[†,‡,¶] Dominykas Lukauskis,[§] Simone Aureli,[†,‡,¶] and Francesco Luigi Gervasio[*,†,‡,§,¶]

†*School of Pharmaceutical Sciences, University of Geneva, Rue Michel-Servet 1, CH-1206 Geneva, CH*

‡*Institute of Pharmaceutical Sciences of Western Switzerland, University of Geneva, CH-1206, Geneva, CH*

¶*Swiss Bioinformatics Institute, University of Geneva, CH-1206, Geneva, CH*

§*Chemistry Department, University College London (UCL), WC1E 6BT, London, UK*

E-mail: francesco.gervasio@unige.ch

[∇]M.K. and A.B. contributed equally to the manuscript.

S1 Systems and system setup

The starting structures of the 11 BRD4-ligand complexes were obtained from Aldeghi and others.[1] Ten of the eleven complexes have crystallographically resolved structures. The PDB IDs used to model these ten complexes are: 4OGI, 3MXF, 4MR3, 4OGJ, 4J0R,

3U5L, 4MR4, 3U5J, 3SVG, 4HBV. One of the eleven complexes lacks an experimentally determined structure; instead, it results from the docking of the ligand to the apo protein (PDB ID: 2OSS[2]) and is modeled based on 3SVG.[1] The 6 initial structures of the Hsp90-ligand complexes were obtained from PDB IDs: 3K99, 3OW6, 3EKO, 2XHT, 2WI2, 2WI3.

The protonation states of the different protein structures at pH 7.4 were determined using the ProteinPrepare via the PlayMolecule web interface.[3] The proteins were solvated with TIP3P water[4] and NaCl was used to match the ionic concentration of the reported experimental settings when available, otherwise physiological NaCl concentration was used. To investigate the effect of different water models on the free energy calculations of BRD4, we also solvated the different BRD4-ligand complexes with the OPC water.[5] The crystallographic waters were retained when present.

The topology of the different ligands was generated with GAFF2[6] and the dihedral energy profiles were re-fitted with the ANI-2X force field[7] via Parameterize on the PlayMolecule web server.[8] Single point charges were calculated using the RESP method.[9] Ligands containing Cl or Br atoms were further parameterized by adding an off-point charge on the halogen atom to properly model the halogen bond.[10]

The various complexes were assembled and parameterized using the LeaP program from AmberTools22. The Amber ff19SB[11] and Amber ff14SB[12] force fields were chosen to parameterize BRD4 and Hsp90 respectively. The Amber topologies were converted to Gromacs topologies using ParmEd.[13]

S2 Unbiased MD

All the molecular dynamics simulations were run with GROMACS 2022[14–21] patched with Plumed-2.9[22–24] The energy of each system was minimised over 50,000 steps using the steepest descent algorithm with a tolerance set at 100 kJ mol^{-1} nm^{-1}. Following

the minimization step, the systems were equilibrated in two steps. First, a 10 ns heating phase was performed in the NVT ensemble, using the V-rescale thermostat ($T = 300$K, $\tau = 0.1$ ps).[25] Two different temperature coupling groups were used, one for the protein and the ligand, and a second one including water molecules and ions. A second 10 ns equilibration phase was then performed in the NPT ensemble using the V-rescale ($T = 300$K, $\tau = 0.5$ ps) and C-rescale ($P = 1$ atm, $\tau = 1$ ps) as thermostat and barostat, respectively.[25,26] The same temperature coupling groups were maintained during the NPT equilibration step. Harmonic restraints were applied to all heavy atoms of the protein and the ligand during both equilibration steps (harmonic constant: 1000 kJ mol^{-1} nm^{-2}). The final structure obtained from the equilibration process served as the starting configuration for the production runs. All systems were simulated in the NPT ensemble with periodic boundary conditions, using the same parameters as in the final equilibration step and removing the harmonic restraints. The particle mesh Ewald method was used to account for long-range electrostatics.[27] A cutoff of 1.2 nm was introduced for short-range electrostatics and Van der Waals interactions. A time step of 2 fs was used for all simulations after constraining the hydrogen stretching modes using the LINCS algorithm.[28]

S3 OneOPES simulations

All OneOPES simulations presented in this work followed a standardized protocol. Specifically, each OneOPES simulation includes eight exchanging replicas following a replica-exchange scheme.[29] All replicas (zero to seven) share the same main OPES Explore bias applied to two primary CVs, namely the protein-ligand contact map and the protein-ligand distance. The barrier height of the main bias was set to 30 kJ mol^{-1} and the bias deposition pace was set to 20000 simulation steps. Replicas one to seven include

additional OPES Explore and OPES MultiThermal components. The additional OPES Explore components act on auxiliary CVs to improve the convergence of replica zero, with an increasing number of auxiliary CVs for higher replicas. The barrier height of the bias deposited on the auxiliary CVs was set to 3 kJ mol^{-1}, with a bias deposition pace of 40000 simulation steps. The initial kernel width, i.e. the sigma parameter, of the various OPES Explore components was defined as the standard deviation of each CV along a 100 ns unbiased MD trajectory of the system. The protocol used to obtain these MD trajectories is described in Section S2. Similarly, higher replicas are subject to an increasingly more aggressive OPES MultiThermal bias, with the target temperature range gradually increasing from 300-302 K (replica one) to 300-370 K (replica seven). Further details on the auxiliary CVs and temperature ranges used can be found in Section S4. An exchange between the eight different replicas was attempted every 2000 simulation steps. The various simulation parameters, such as thermostat temperature and reference pressure, were controlled as described in Section S2. Finally, all OneOPES simulations include a funnel-shaped wall and a C_α-RMSD constraint.

We used a funnel-shaped wall as previously reported.[30–32] Two sets of atoms within stable protein regions were defined to specify the origin and direction of the funnel axis. The specific atoms used are listed in the provided PLUMED files (Data and Software Availability Section). Harmonic restraints, referred to as upper and lower walls, were applied at both ends of the funnel axis to confine the ligand's exploration within the funnel-shaped restraint. The upper wall, positioned distally from the protein binding site, was located to ensure a sufficient distance between any protein and ligand atom, allowing the ligand to sample configurations in bulk water without interacting with the protein.

To ensure the structural stability of the proteins despite the presence of external bias potentials, an RMSD harmonic wall was applied to the C_α of the protein backbone. In

addition, for the simulations of BRD4, RMSD walls were also applied to the most flexible regions of the protein to ensure its stability. Specifically, we constrained the distance between the following residue pairs: SER42-THR60,GLN78-ASN93,ASN93-ASP106 and MET132-ALA152. The upper limits for all walls were determined by examining the fluctuations of the C_α RMSD and the selected contacts during unbiased simulations of 100 ns. This choice was made to allow the protein structure to fluctuate and adjust to bind the ligand while avoiding local or global unfolding.

S4 Auxiliary CVs

S4.1 BRD4

We included the following auxiliary CVs in the presented OneOPES simulations of BRD4. Additional information on the specif atoms selected are included in the plumed file for each protein-ligand complex (See Data and Software Availability section in the main text).

- **water-ligand (wl):** 3 water coordination sites, namely wl1-3, located on geometrically distant polar atoms of the ligand;
- **water-protein (wp):** 4 water coordination sites, namely wp1-4, located on geometrically distant polar atoms of the binding pocket, for BRD4 they are located on residues CYS95, TYR98, TYR56, MET91;
- **ligand torsions (tors):** rotatable bonds of a given ligand associated with higher energy barriers;

The main CVs (CMAP and ligand distance from binding site, the auxiliary CVs and the temperature ranges were combined in our OneOPES simulations as shown in the

following Table:

The different CVs and temperatures used for the OneOPES simulations of BRD4. On the rows *Main CVs*, *Auxiliary CV wp* , *Auxiliary CV wl*, and *Auxiliary CV tors* we report how the CVs have been combined in each replica. On the row *OPES MultiThermal* we display the highest selected temperature for each replica in K. Each replica spans the temperature range defined by the thermostat, i.e. 300 K and the selected OPES Multithermal temperature. The auxiliary CVs for the torsional angles are system dependent, further information can be found in the PLUMED file of each system (see the Data and Software Availability section in the main text).

**Replicas**	**0**	**1**	**2**	**3**	**4**	**5**	**6**	**7**
Main CVs	yes	yes	yes	yes	yes	yes	yes	yes
Auxiliary CV wp	no	wp1	wp1-2	wp1-3	wp1-4	wp1-4	wp1-4	wp1-4
Auxiliary CV wl	no	no	no	no	no	wl1	wl1-2	wl1-3
Auxiliary CV tors	no	yes	yes	yes	yes	yes	yes	yes
OPES MultiThermal	no	302	305	310	320	335	350	370

S4.2 HSP90

We included the following auxiliary CVs in the presented OneOPES simulations of Hsp90. Additional information on the specif atoms selected are included in the plumed file for each protein-ligand complex (See Data and Software Availability section in the main text).

- **water-ligand (wl):** 2 water coordination sites, namely wl1-2, located on geometrically distant polar atoms of the ligand;
- **water-protein (wp):** 4 water coordination sites, namely wp1-4, located on geometrically distant polar atoms of the binding pocket, for Hsp90 they are located on residues ASN36, ASP78, ASP87, THR169;
- **ligand torsions (tors):** rotatable bonds of a given ligand associated with higher energy barriers;

The main CVs (CMAP and ligand distance from binding site, the auxiliary CVs and the temperature ranges were combined in our OneOPES simulations as shown in following Table:

The different CVs and temperatures used for the OneOPES simulations of Hsp90. On the rows *Main CVs*, *Auxiliary CV wp* , *Auxiliary CV wl*, and *Auxiliary CV tors* we report how the CVs have been combined in each replica. On the row *OPES MultiThermal* we display the highest selected temperature for each replica in K. Each replica spans the temperature range defined by the thermostat, i.e. 300 K and the selected OPES Multithermal temperature. The auxiliary CVs for the torsional angles are system dependent, further information can be found in the PLUMED file of each system (see the Data and Software Availability section in the main text).

**Replicas**	**0**	**1**	**2**	**3**	**4**	**5**	**6**	**7**
Main CVs	yes	yes	yes	yes	yes	yes	yes	yes
Auxiliary CV wp	no	wp1	wp1-2	wp1-3	wp1-4	wp1-4	wp1-4	wp1-4
Auxiliary CV wl	no	no	no	no	no	wl1	wl1-2	wl1-2
Auxiliary CV tors	no	yes	yes	yes	yes	yes	yes	yes
OPES MultiThermal	no	302	305	310	320	335	350	370

S5 Free energies and error estimation

For each system, we identified ligand-bound and ligand-unbound states based on the distance of the ligand to the binding site. Accounting for the entropy loss introduced by the funnel restraint[30,31], we calculated the binding free energy difference (ΔG) as follows:

$$\Delta G = -k_{\mathrm{B}} T \log \left(C^0 \pi R_{\mathrm{cyl}}^2 \int_{\mathrm{B}} \mathrm{d}z \, \exp\left(-\beta(F(z) - F_{\mathrm{U}})\right) \right) \tag{1}$$

where k_{B} is Boltzmann's constant, $T = 300$ K is the system's temperature, $\beta = 1/(k_{\mathrm{B}}T)$, $C^0 = 1/1660\text{Å}^{-3}$ the standard concentration, z the position of the ligand's center of mass along the funnel's axis, $F(z)$ is the free energy value along the funnel axis and F_{U} its reference value in the unbound state.

When calculating the binding free energy from OneOPES simulations, it is crucial to remove the initial out-of-equilibrium portions that present large fluctuations in the deposited bias, which would negatively affect the reweighting procedure. To correctly identify and exclude such regions, we have adopted the following strategy (Fig. S6). First, on replica zero, we monitor the OPES Explore bias as a function of simulation time for both the ligand-bound and ligand-unbound states. We then calculate the rolling average of the deposited bias profile with a window size of 0.5 ns and subsequently determine its derivative. Bias regions where the time derivative is close to zero indicate that the simulations have approached a quasi-static regime. We analyze the distribution of the smoothed bias derivative values and identify outliers. Outliers are defined as derivative values with a z-score greater than 3 from the distribution, indicating values significantly higher than the mean.

Next, we scan the bias profile in 100 ns windows to identify the first window with no derivative value greater than the smallest outlier, marking the start of the plateau region of the simulation. We repeat this analysis for both the ligand-bound and ligand-unbound states and identify the simulation time corresponding to the start of the plateau region in the two bias profiles. We select the largest of the two and discard configurations sampled before the start of this plateau region. The equilibration times obtained for each system are listed in Tables S4-S5.

The error estimates are calculated via block analysis. After discarding the first equilibration phase, we divide the remaining simulation into an increasing number of blocks, ranging from three to nine blocks in total. The maximum error obtained from these sets of blocks is taken as the presented free energy uncertainty. Additionally, the error estimate is also used to stop the simulations, following an error-informed stopping strategy. We reasoned that if the error associated with the free energy estimates remains within a given value (1 kcal mol^{-1}) for a sufficiently long simulation time, we have achieved a

good estimate of the binding free energy and can therefore stop the simulation. Specifically, we calculate the uncertainty of the free energy for increasing simulation times, with increments of 100 ns. When three consecutive increments show an error estimate within 1 kcal mol^{-1}, indicating a stable error for at least 300 ns of simulation, we stop the run (Fig. S7). Nevertheless, we also analyzed the errors at longer simulation times to examine how the duration of the simulation affects the free energy estimates. An example is shown in Fig. S4.

Additionally, we compared the resulting error estimation obtained with the above-mentioned strategy and the error taken as the standard deviation of three independent OneOPES simulations of the same system. Notably, the two error estimates converged to the same value, further indicating that our error-informed stopping strategy retrieves accurate error estimates (Fig. S8).

Table S1: **Summary of the BRD4 binding free energy results using OneOPES.** ΔG_{calc}^{OPC} represents the calculated standard binding free energy with the OPC water model; ΔG_{exp} denotes the experimental standard binding free energy, with references provided. The PDB files used as input are listed. Errors in the experimental measurements are reported as one standard deviation, where available. Errors for the calculated standard free energies were obtained using block analysis. All values are given in kcal mol^{-1}. The simulation time for each replica (eight exchanging replicas per simulation) is reported.

Compound	ΔG_{calc}^{OPC}	ΔG_{exp}	ΔG_{calc}^{OPC} - ΔG_{exp}	PDB	μs per replica
1	-11.2 ± 0.3	-9.8 ± 0.1[33]	-1.4	4OGI	1.3
2	-9.0 ± 0.3	-9.6 ± 0.1[34]	0.6	3MXF	0.7
3	-9.1 ± 0.4	-9.0 ± 0.1[35]	-0.1	4MR3	0.5
4	-11.8 ± 0.2	-8.9 ± 0.1[33]	-2.9	4OGJ	0.6
5	-10.0 ± 0.8	-8.8 ± 0.1[36]	-1.2	4J0R	0.5
6	-10.3 ± 0.4	-8.2 ± 0.1[37]	-2.1	3U5L	0.5
7	-8.0 ± 0.3	-7.8 ± 0.1[35]	-0.2	4MR4	0.5
8	-8.8 ± 0.5	-7.4 ± 0.1[37]	-1.4	3U5J	0.5
9	-9.4 ± 0.8	-7.3 ± 0.0[36]	-2.1	3SVG	0.5
10	-6.6 ± 0.4	-6.3 ± 0.1[38]	-0.3	4HBV	0.5
11	-9.7 ± 0.4	-5.6[39]	-4.1	Model	0.6

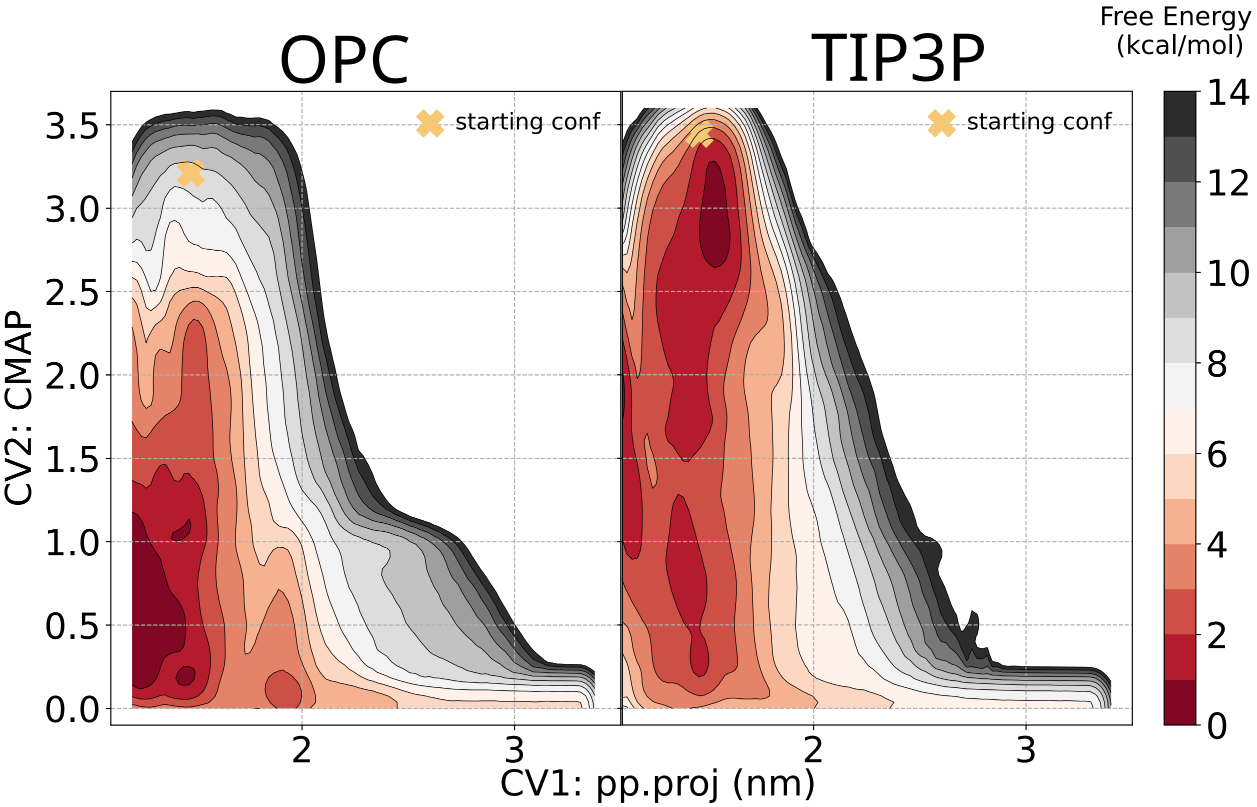

Figure S1: **The impact of water models: 2D free energy surfaces (FES) of the BRD4 and ligand 10 complex.** *Left:* 2D FES obtained with OPC water using the presented OneOPES strategy. *Right:* 2D FES obtained with TIP3P water using the presented OneOPES strategy. The yellow cross indicates the initial crystallographic pose of ligand 10, plotted as a function of the two main collective variables (CVs) used in OneOPES. The crystallographic minimum is not stable when OPC is used as the water model, whereas it corresponds to the main free energy minimum with TIP3P water. The simulation time and parameters were the same for both the OPC and TIP3P OneOPES runs.

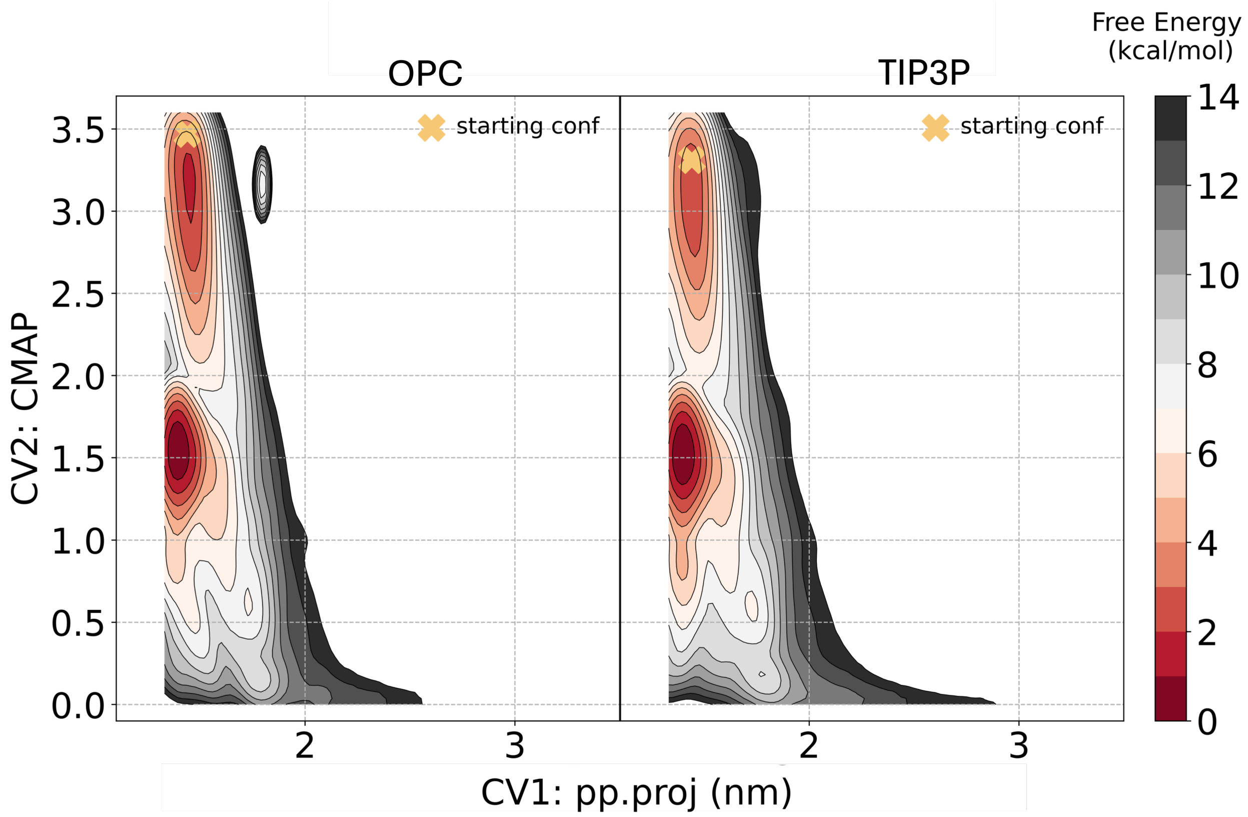

Figure S2: **Effect of force field biases: 2D free energy surfaces (FES) of the BRD4 and ligand 6 complex.** *Left:* 2D FES obtained with OPC water using the presented OneOPES strategy. *Right:* 2D FES obtained with TIP3P water using the presented OneOPES strategy. The yellow cross indicates the initial crystallographic pose of ligand 6, plotted as a function of the two main collective variables (CVs) used in OneOPES. Although the deepest free energy minimum does not align with the crystallographic pose in both cases, the crystallographic binding mode is identified as a local minimum on the free energy surface.

Table S2: **Effect of force field biases on binding free energy estimates.** The binding free energy of ligand 6 is reported for two scenarios: considering only crystallographic-like states (*6 crystal-like*) and including both force field-assigned minima (*6 force field minima*). When both force field assigned minima are included in the estimate, the observed deviation from experimentally determined values is significantly higher. ΔG_{calc}^{TIP3P} represents the calculated standard binding free energy with the TIP3P water model; ΔG_{exp} denotes the experimental standard binding free energy, with references provided. The PDB files used as input are listed. Errors in the experimental measurements are reported as one standard deviation, where available. Errors for the calculated standard free energies were obtained using block analysis. All values are given in kcal mol^{-1}. The simulation time for each replica (eight exchanging replicas per simulation) is reported.

Compound 6	ΔG_{calc}^{TIP3P}	ΔG_{exp}	ΔG_{calc}^{TIP3P} - ΔG_{exp}	PDB	μs per replica
crystal-like	-10 $\pm$ 0.3	-8.2 $\pm$ 0.1[37]	-1.8 $\pm$ 0.3	3U5L	1.4
force field minima	-10.7 $\pm$ 0.4	-8.2 $\pm$ 0.1[37]	-2.5 $\pm$ 0.4	3U5L	0.5

S6 MetaDynamics simulations

Single-replica Funnel-restrained Well-tempered Metadynamics (FunMetaD) was used to calculate the binding free energies of the systems presented. The CVs used are the same as the two primary CVs used in the OneOPES runs. Gaussian hills were deposited every 1000 integration steps with an initial height of 1.5 kJ mol^{-1} with a bias factor of 10. The Gaussian sigma was set to 0.15 nm and 0.07 nm for the protein-ligand contact map and protein-ligand distance CVs, respectively. The FunMetaD simulations were terminated when a thorough exploration of the relevant CV space was achieved and the binding free energy estimates adopted an asymptotic behaviour. Each FunMetaD simulation was run in triplicate, i.e. three independent replicas, for error estimation. All FunMetaD simulations include the same funnel-shaped and a C_α-RMSD walls as the one used for OneOPES.

Table S3: **Summary of the BRD4 binding free energy results using Funnel-restrained Well-Tempered Metadynamics.** $\Delta G_{calc}^{FunMetaD}$ represents the calculated standard binding free energy with the TIP3P water model; ΔG_{exp} denotes the experimental standard binding free energy, with references provided. The PDB files used as input are listed. Errors in the experimental measurements are reported as one standard deviation, where available. Errors for the calculated standard free energies were obtained using three independent well-tempered Metadynamics replicas. All values are given in kcal mol^{-1}. The simulation time for each replica (three independent replicas per calculation) is reported.

Compound	$\Delta G_{calc}^{FunMetaD}$	ΔG_{exp}	$\Delta G_{calc}^{FunMetaD}$ - ΔG_{exp}	PDB	μs per replica
1	-9.1 ± 2.4	-9.8 ± 0.1[33]	0.7	4OGI	1.0
2	-7.9 ± 1.1	-9.6 ± 0.1[34]	1.7	3MXF	1.0
3	-7.0 ± 1.5	-9.0 ± 0.1[35]	2.0	4MR3	1.0
4	-6.3 ± 0.2	-8.9 ± 0.1[33]	2.6	4OGJ	1.0
5	-5.7 ± 2.1	-8.8 ± 0.1[36]	3.1	4J0R	1.0
6	-9.0 ± 2.3	-8.2 ± 0.1[37]	-0.8	3U5L	1.0
7	-6.7 ± 1.5	-7.8 ± 0.1[35]	1.1	4MR4	1.0
8	-8.6 ± 0.9	-7.4 ± 0.1[37]	-1.2	3U5J	1.0
9	-6.6 ± 2.0	-7.3 ± 0.0[36]	0.7	3SVG	1.0
10	-3.9 ± 0.8	-6.3 ± 0.1[38]	2.4	4HBV	1.0
11	-3.2 ± 1.6	-5.6[39]	2.4	Model	1.0

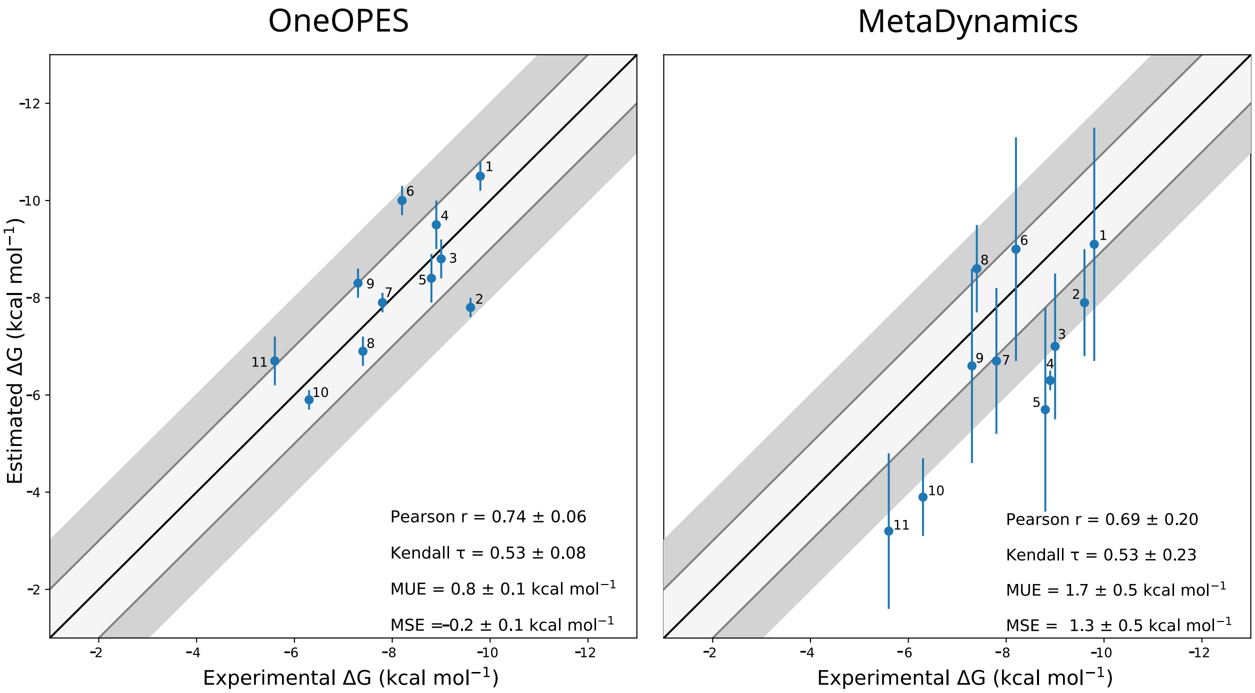

Figure S3: **Free energy correlation plots of the 11 BRD4-ligand complexes obtained with either OneOPES or FunMetaD simulations.** *Left*: results obtained with OneOPES. Errors are computed using block analysis. *Right*: results obtained with Funnel-restrained Well-tempered Metadynamics. Errors are calculated as the standard deviation of three independent simulations. The dark gray shaded area represents a deviation of ± 2 kcal mol^{-1} from the experimental values, while the light gray corresponds to a deviation of ± 1 kcal mol^{-1}. The ideal correlation is shown as a black line. The correlation plots are shown as experimental versus calculated binding free energy values.

Table S4: **Bias equilibration times for the presented BRD4-ligand OneOPES simulations**

Compound	Equilibration phase (μs)	PDB
1	0.2	4OGI
2	0.4	3MXF
3	0.2	4MR3
4	0.3	4OGJ
5	0.2	4J0R
6	0.2	3U5L
7	0.2	4MR4
8	0.2	3U5J
9	0.2	3SVG
10	0.4	4HBV
11	0.2	Model

Table S5: **Bias equilibration times for the presented Hsp90-ligand OneOPES simulations**

Compound	Equilibration phase (μs)	PDB
1	0.2	3K99
2	0.2	3OW6
3	0.3	3EKO
4	0.2	2XHT
5a	0.2	2WI2
5b	0.2	2WI3

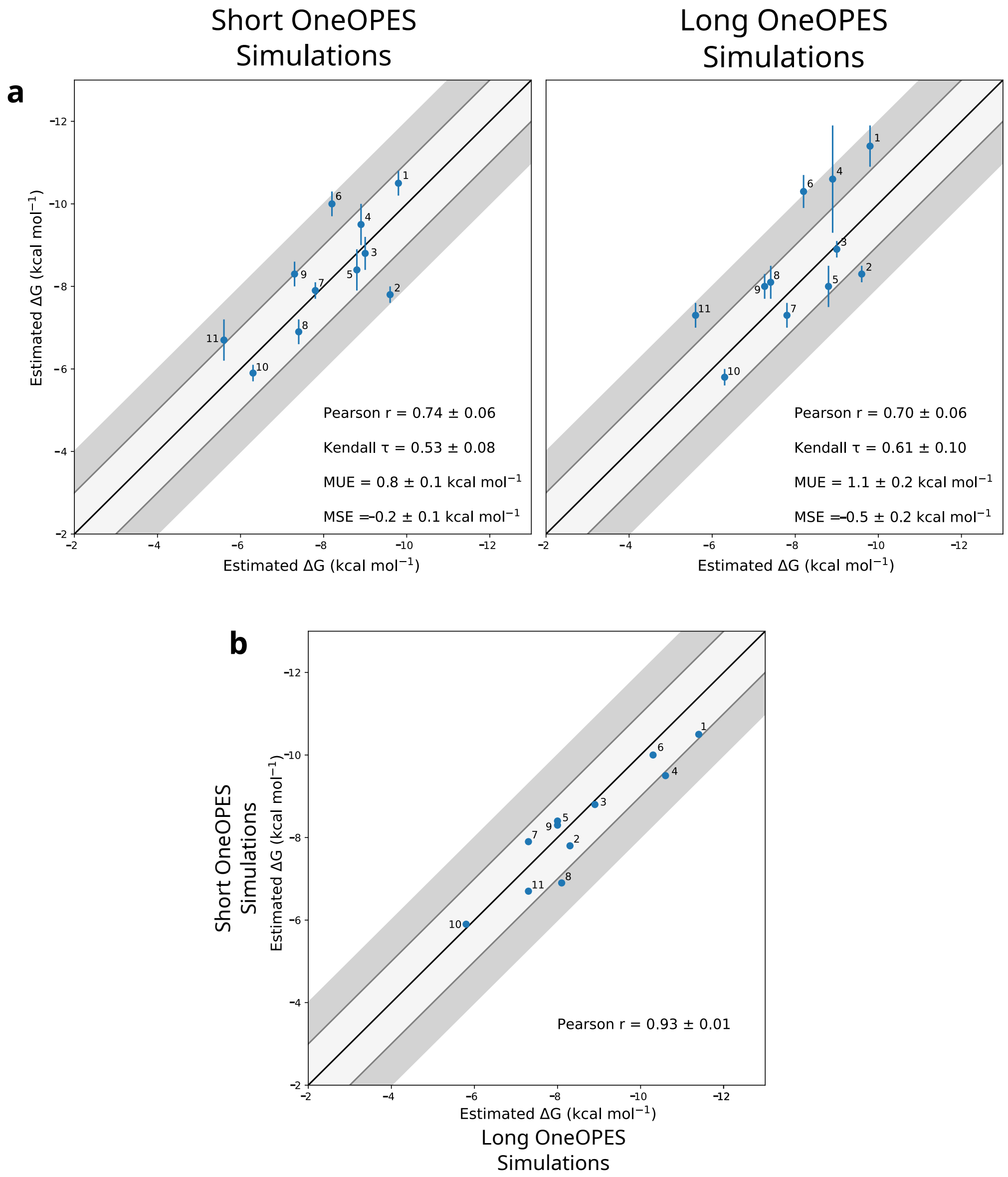

Figure S4: **Comparison of short vs. long OneOPES simulations of BRD4-ligand complexes.** ***a)*** Correlation plots of experimental versus calculated binding free energies obtained using short (left) or long (right) OneOPES simulations. The dark gray shaded area represents a deviation of ± 2 kcal mol^{-1} from the experimental values, while the light gray corresponds to a deviation of ± 1 kcal mol^{-1}. The ideal correlation is shown as a black line. ***b)*** Correlation plot between the results obtained with short and long OneOPES simulations. The results show a strong correlation between the two sets of simulations. The simulation time for the long OneOPES simulations is 2 μs per replica. Details of the different simulation times for the short OneOPES simulations are given in Table 1 of the main text.

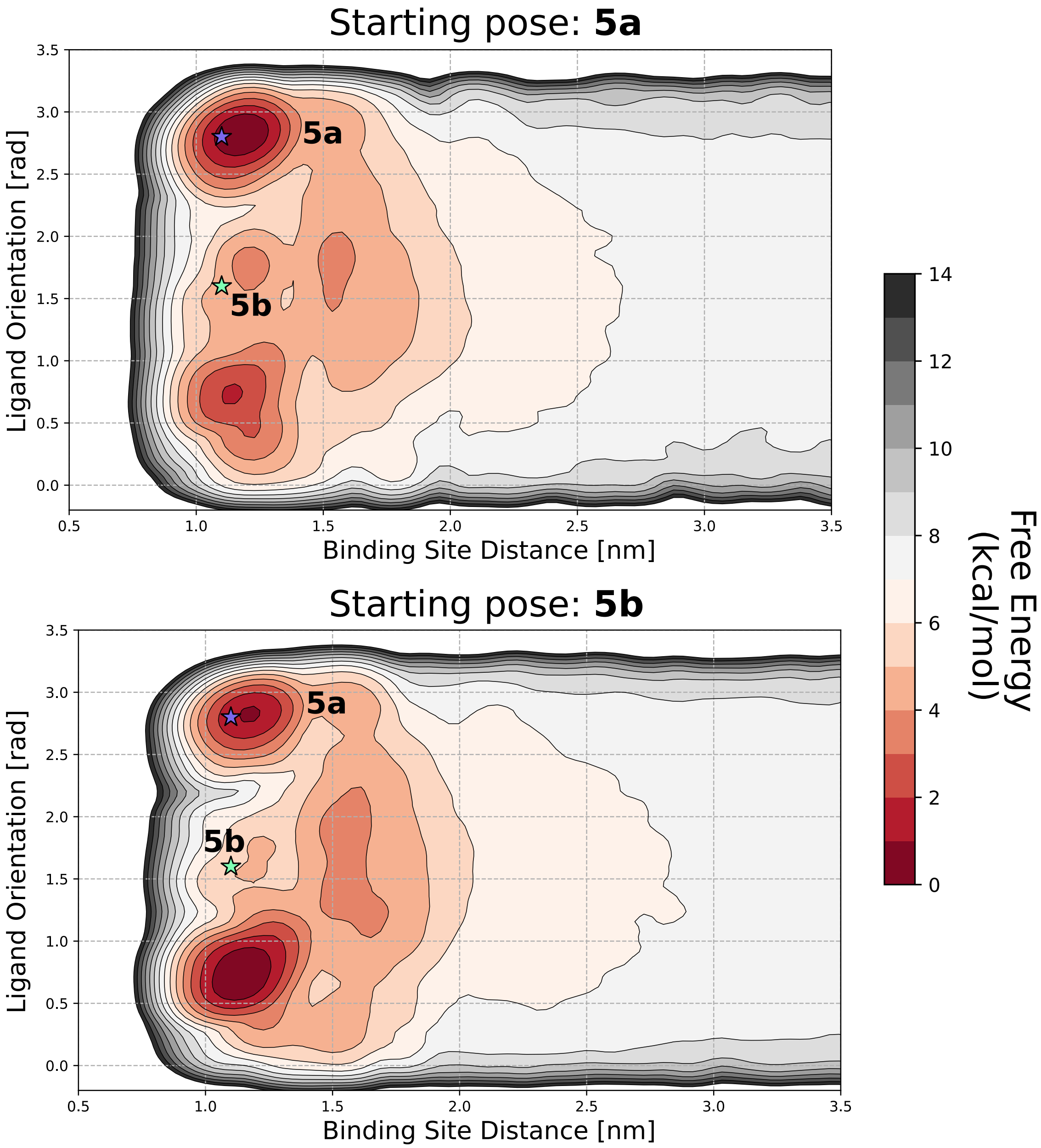

Figure S5: **Sampling of different ligand binding modes with OneOPES.** *Top:* 2D free energy surface obtained with a 1.75 μs-long OneOPES simulation of ligand 5 stargin from the binding pose 5a. *Bottom:* 2D free energy surface obtained with a 1.75 μs-long OneOPES simulation of ligand 5 starting from the binding pose 5b. The binding free energy is plotted as a function of the ligand's distance from the binding site and its orientation. The minima corresponding to the two different binding modes are marked by purple and green stars, representing pose 5a and pose 5b, respectively. These 2D projections illustrate how longer OneOPES simulations converge to the same free energy landscape, regardless of the initial binding mode.

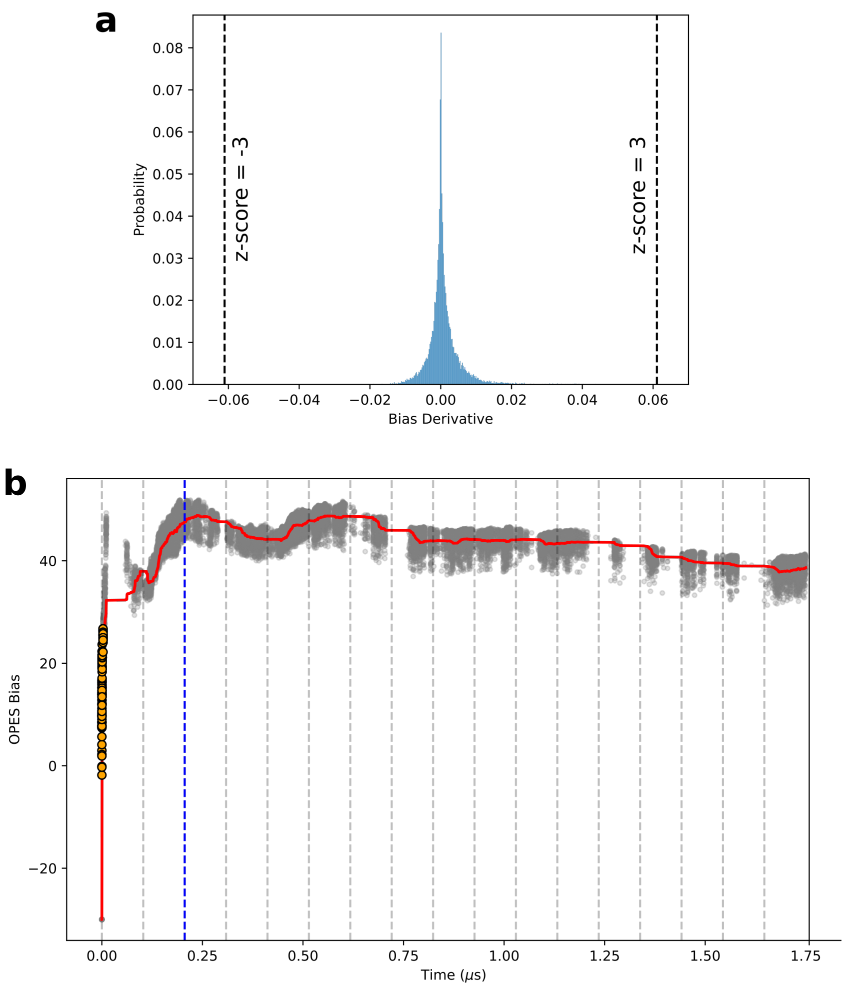

Figure S6: **Example of bias equilibration in OneOPES simulations.** ***a)*** Derivatives distribution of the rolling-averaged main OneOPES bias as a function of simulation time (window size of 0.5 ns). The z-score thresholds, above which a derivative is considered an outlier, are indicated by the black dashed lines. We effectively selected only values above 3, as we considered the absolute value of the derivatives. ***b)*** Given the value of the smallest outlier, we define the start of the plateau region as the time corresponding to the end of the first 100 ns window that does not contain any outliers. The main OneOPES bias is shown as gray dots, with the rolling-averaged bias depicted as a red line. The 100 ns windows are marked by vertical dashed lines. Bias values whose time derivatives are considered outliers are indicated as yellow dots, and the start of the plateau region is denoted by a vertical dashed blue line.

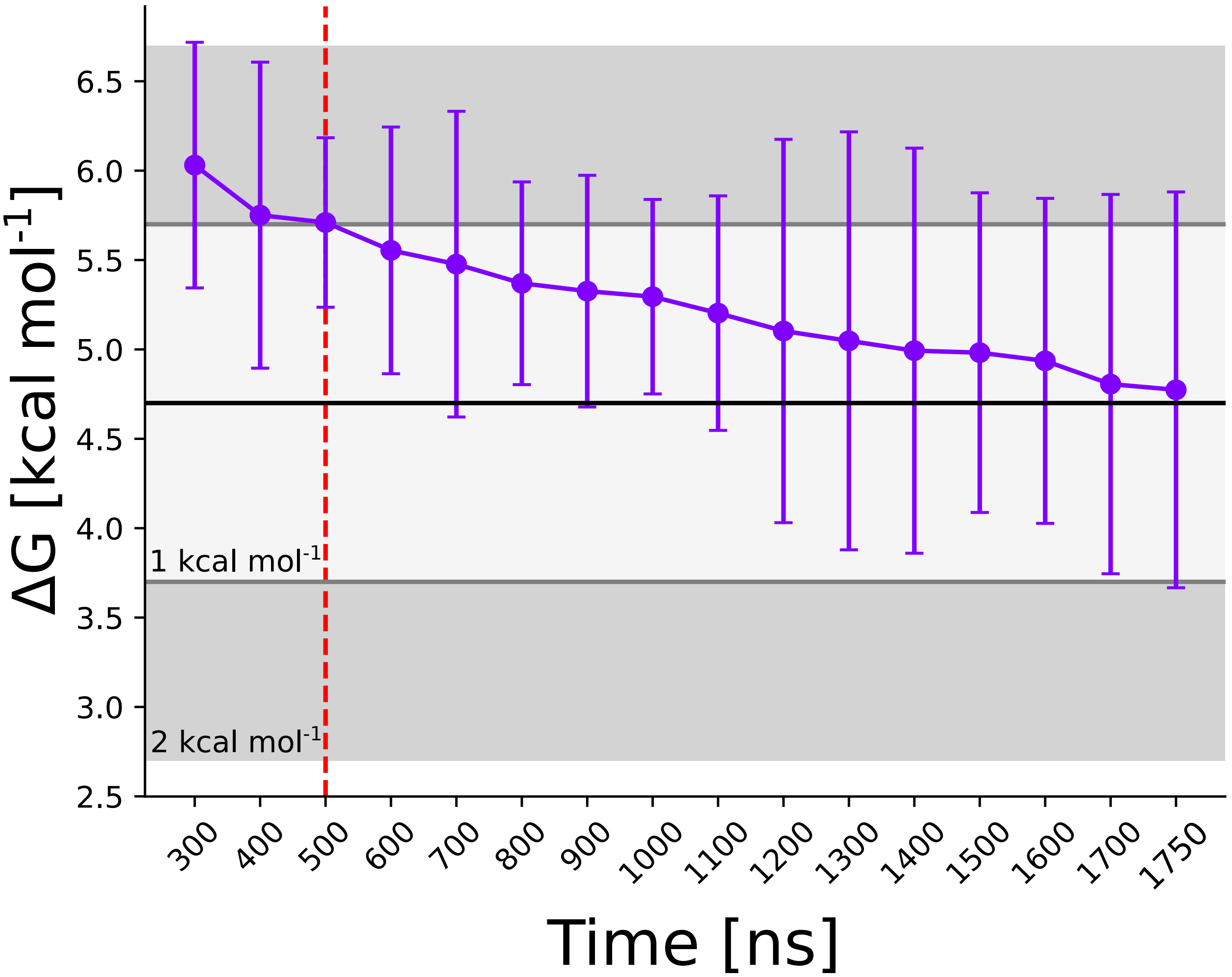

Figure S7: **Example of the presented error-informed stopping strategy.** Binding free energy as a function of simulation time for the Hsp90 and ligand 5a complex. After discarding the initial equilibration phase, we calculated the binding free energy and the relative uncertainty at 100 ns increments. The dashed red line indicates the simulation time when we considered the uncertainty measure converged, i.e., after three consecutive increments with an uncertainty estimate within 1 kcal mol^{-1}. The free energy uncertainty was calculated with block analysis. The dark gray shaded area represents a deviation of ±2 kcal mol^{-1} from the experimental values, while the light gray corresponds to a deviation of ±1 kcal mol^{-1}. The experimental binding free energy is shown as a black line.

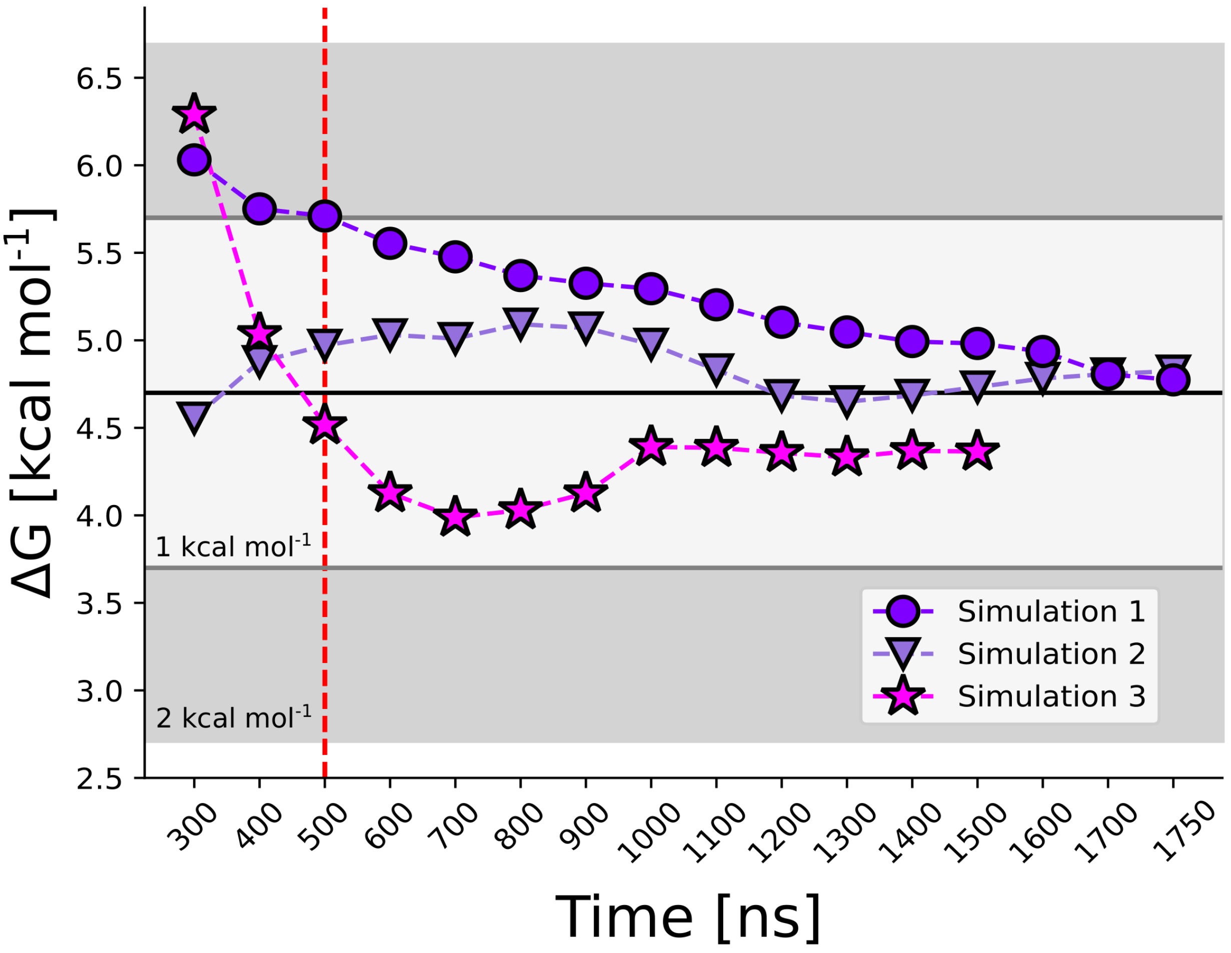

Figure S8: **Three independent OneOPES simulations of Hsp90 and ligand 5a complex.** Binding free energy as a function of simulation time for three independent simulations of the same protein-ligand complex. After an equilibration phase, the three simulations converge to comparable binding free energy estimates both at early simulation times (red dashed line) as well as at longer simulation times. The error estimate calculated at 500 ns, as the standard deviation of the three independent runs, is 0.5 kcal mol^{-1}, comparable to the uncertainty estimation obtained at 500 ns for each independent run calculated with block analysis (Table S6). The dark gray shaded area represents a deviation of ± 2 kcal mol^{-1} from the experimental values, while the light gray corresponds to a deviation of ± 1 kcal mol^{-1}. The experimental binding free energy is shown as a black line.

Table S6: **Comparison between error estimations.** ΔG_{calc} represents the calculated standard binding free energy at 500 ns for three independent simulations of the Hsp90 and ligand 5a complex. Errors for the calculated standard free energies were obtained using either block analysis for each simulation or as the standard deviation of the three independent measures. ΔG_{exp} denotes the experimental standard binding free energy, with references provided. All values are given in kcal mol^{-1}.

Compound 5a	ΔG_{calc}	Error with block analysis	Error as SD	ΔG_{exp}	ΔG_{calc} - ΔG_{exp}
Simulation 1	-5.7	± 0.5	± 0.5	-4.7 [40]	-1.0
Simulation 2	-5.0	± 0.4	± 0.5	-4.7 [40]	-0.3
Simulation 3	-4.5	± 1.0	± 0.5	-4.7 [40]	0.2

References

(1) Aldeghi, M.; Heifetz, A.; Bodkin, M. J.; Knapp, S.; Biggin, P. C. Accurate calculation of the absolute free energy of binding for drug molecules. *Chemical Science* **2016**, *7*, 207–218.

(2) Filippakopoulos, P.; Picaud, S.; Mangos, M.; Keates, T.; Lambert, J.-P.; Barsyte-Lovejoy, D.; Felletar, I.; Volkmer, R.; Müller, S.; Pawson, T.; others Histone recognition and large-scale structural analysis of the human bromodomain family. *Cell* **2012**, *149*, 214–231.

(3) Martínez-Rosell, G.; Giorgino, T.; De Fabritiis, G. PlayMolecule ProteinPrepare: a web application for protein preparation for molecular dynamics simulations. *Journal of chemical information and modeling* **2017**, *57*, 1511–1516.

(4) Jorgensen, W. L.; Chandrasekhar, J.; Madura, J. D.; Impey, R. W.; Klein, M. L. Comparison of simple potential functions for simulating liquid water. *The Journal of Chemical Physics* **1983**, *79*, 926–935.

(5) Izadi, S.; Anandakrishnan, R.; Onufriev, A. V. Building Water Models: A Different Approach. *The Journal of Physical Chemistry Letters* **2014**, *5*, 3863–3871.

(6) Wang, J.; Wolf, R. M.; Caldwell, J. W.; Kollman, P. A.; Case, D. A. Development and testing of a general amber force field. *Journal of Computational Chemistry* **2004**, *25*, 1157–1174.

(7) Devereux, C.; Smith, J. S.; Huddleston, K. K.; Barros, K.; Zubatyuk, R.; Isayev, O.; Roitberg, A. E. Extending the Applicability of the ANI Deep Learning Molecular Potential to Sulfur and Halogens. *Journal of Chemical Theory and Computation* **2020**, *16*, 4192–4202.

(8) Galvelis, R.; Doerr, S.; Damas, J. M.; Harvey, M. J.; De Fabritiis, G. A Scalable Molecular Force Field Parameterization Method Based on Density Functional Theory and Quantum-Level Machine Learning. *Journal of Chemical Information and Modeling* **2019**, *59*, 3485–3493.

(9) Bayly, C. I.; Cieplak, P.; Cornell, W.; Kollman, P. A. A well-behaved electrostatic potential based method using charge restraints for deriving atomic charges: the RESP model. *The Journal of Physical Chemistry* **1993**, *97*, 10269–10280.

(10) Ibrahim, M. A. A. Molecular mechanical study of halogen bonding in drug discovery. *Journal of Computational Chemistry* **2011**, *32*, 2564–2574.

(11) Tian, C.; Kasavajhala, K.; Belfon, K. A. A.; Raguette, L.; Huang, H.; Migues, A. N.; Bickel, J.; Wang, Y.; Pincay, J.; Wu, Q.; Simmerling, C. ff19SB: Amino-Acid-Specific Protein Backbone Parameters Trained against Quantum Mechanics Energy Surfaces in Solution. *Journal of Chemical Theory and Computation* **2020**, *16*, 528–552.

(12) Maier, J. A.; Martinez, C.; Kasavajhala, K.; Wickstrom, L.; Hauser, K. E.; Simmerling, C. ff14SB: Improving the Accuracy of Protein Side Chain and Backbone Parameters from ff99SB. *Journal of Chemical Theory and Computation* **2015**, *11*, 3696–3713.

(13) Shirts, M. R.; Klein, C.; Swails, J. M.; Yin, J.; Gilson, M. K.; Mobley, D. L.; Case, D. A.; Zhong, E. D. Lessons learned from comparing molecular dynamics engines on the SAMPL5 dataset. *Journal of computer-aided molecular design* **2017**, *31*, 147–161.

(14) Páll, S.; Zhmurov, A.; Bauer, P.; Abraham, M.; Lundborg, M.; Gray, A.; Hess, B.; Lindahl, E. Heterogeneous parallelization and acceleration of molecular dynamics simulations in GROMACS. *The Journal of Chemical Physics* **2020**, *153*, 134110.

(15) Abraham, M. J.; Murtola, T.; Schulz, R.; Páll, S.; Smith, J. C.; Hess, B.; Lindahl, E.

GROMACS: High performance molecular simulations through multi-level parallelism from laptops to supercomputers. *SoftwareX* **2015**, *1-2*, 19–25.

(16) Páll, S.; Abraham, M. J.; Kutzner, C.; Hess, B.; Lindahl, E. In *Solving Software Challenges for Exascale*; Markidis, S., , Laure, E., Eds.; Springer International Publishing, 2015; pp 3–27.

(17) Pronk, S.; Páll, S.; Schulz, R.; Larsson, P.; Bjelkmar, P.; Apostolov, R.; Shirts, M. R.; Smith, J. C.; Kasson, P. M.; van der Spoel, D.; Hess, B.; Lindahl, E. GROMACS 4.5: a high-throughput and highly parallel open source molecular simulation toolkit. *Bioinformatics* **2013**, *29*, 845–854.

(18) Hess, B.; Kutzner, C.; van der Spoel, D.; Lindahl, E. GROMACS 4: Algorithms for Highly Efficient, Load-Balanced, and Scalable Molecular Simulation. *Journal of Chemical Theory and Computation* **2008**, *4*, 435–447.

(19) Van Der Spoel, D.; Lindahl, E.; Hess, B.; Groenhof, G.; Mark, A. E.; Berendsen, H. J. C. GROMACS: Fast, flexible, and free. *Journal of Computational Chemistry* **2005**, *26*, 1701–1718.

(20) Lindahl, E.; Hess, B.; van der Spoel, D. GROMACS 3.0: a package for molecular simulation and trajectory analysis. *Journal of Molecular Modeling* **2001**, *7*, 306–317.

(21) Berendsen, H.; van der Spoel, D.; van Drunen, R. GROMACS: A message-passing parallel molecular dynamics implementation. *Computer Physics Communications* **1995**, *91*, 43–56.

(22) Bonomi, M.; Bussi, G.; Camilloni, C.; Tribello, G. A.; Banáš, P.; Barducci, A.; Bernetti, M.; Bolhuis, P. G.; Bottaro, S.; Branduardi, D.; Capelli, R.; Carloni, P.; Ceriotti, M.; Cesari, A.; Chen, H.; Chen, W.; Colizzi, F.; De, S.; De La Pierre, M.;

Donadio, D.; Drobot, V.; Ensing, B.; Ferguson, A. L.; Filizola, M.; Fraser, J. S.; Fu, H.; Gasparotto, P.; Gervasio, F. L.; Giberti, F.; Gil-Ley, A.; Giorgino, T.; Heller, G. T.; Hocky, G. M.; Iannuzzi, M.; Invernizzi, M.; Jelfs, K. E.; Jussupow, A.; Kirilin, E.; Laio, A.; Limongelli, V.; Lindorff-Larsen, K.; Löhr, T.; Marinelli, F.; Martin-Samos, L.; Masetti, M.; Meyer, R.; Michaelides, A.; Molteni, C.; Morishita, T.; Nava, M.; Paissoni, C.; Papaleo, E.; Parrinello, M.; Pfaendtner, J.; Piaggi, P.; Piccini, G.; Pietropaolo, A.; Pietrucci, F.; Pipolo, S.; Provasi, D.; Quigley, D.; Raiteri, P.; Raniolo, S.; Rydzewski, J.; Salvalaglio, M.; Sosso, G. C.; Spiwok, V.; Šponer, J.; Swenson, D. W. H.; Tiwary, P.; Valsson, O.; Vendruscolo, M.; Voth, G. A.; White, A.; consortium, T. P. Promoting transparency and reproducibility in enhanced molecular simulations. *Nature Methods* **2019**, *16*, 670–673.

(23) Tribello, G. A.; Bonomi, M.; Branduardi, D.; Camilloni, C.; Bussi, G. PLUMED 2: New feathers for an old bird. *Computer Physics Communications* **2014**, *185*, 604–613.

(24) Bonomi, M.; Branduardi, D.; Bussi, G.; Camilloni, C.; Provasi, D.; Raiteri, P.; Donadio, D.; Marinelli, F.; Pietrucci, F.; Broglia, R. A.; Parrinello, M. PLUMED: A portable plugin for free-energy calculations with molecular dynamics. *Computer Physics Communications* **2009**, *180*, 1961–1972.

(25) Bussi, G.; Donadio, D.; Parrinello, M. Canonical sampling through velocity rescaling. *The Journal of Chemical Physics* **2007**, *126*, 014101.

(26) Bernetti, M.; Bussi, G. Pressure control using stochastic cell rescaling. *The Journal of Chemical Physics* **2020**, *153*, 114107.

(27) Darden, T.; York, D.; Pedersen, L. Particle mesh Ewald: An N log(N) method for Ewald sums in large systems. *The Journal of Chemical Physics* **1993**, *98*, 10089–10092.

(28) Hess, B.; Bekker, H.; Berendsen, H. J. C.; Fraaije, J. G. E. M. LINCS: A linear constraint solver for molecular simulations. *Journal of Computational Chemistry* **1997**, *18*, 1463–1472.

(29) Bussi, G. Hamiltonian replica exchange in GROMACS: a flexible implementation. *Molecular Physics* **2014**, *112*, 379–384.

(30) Deng, Y.; Roux, B. Computations of standard binding free energies with molecular dynamics simulations. *The Journal of Physical Chemistry B* **2009**, *113*, 2234–2246.

(31) Limongelli, V.; Bonomi, M.; Parrinello, M. Funnel metadynamics as accurate binding free-energy method. *Proceedings of the National Academy of Sciences* **2013**, *110*, 6358–6363.

(32) Evans, R.; Hovan, L.; Tribello, G. A.; Cossins, B. P.; Estarellas, C.; Gervasio, F. L. Combining Machine Learning and Enhanced Sampling Techniques for Efficient and Accurate Calculation of Absolute Binding Free Energies. *Journal of Chemical Theory and Computation* **2020**, *16*, 4641–4654.

(33) Ciceri, P.; Müller, S.; O'Mahony, A.; Fedorov, O.; Filippakopoulos, P.; Hunt, J. P.; Lasater, E. A.; Pallares, G.; Picaud, S.; Wells, C.; Martin, S.; Wodicka, L. M.; Shah, N. P.; Treiber, D. K.; Knapp, S. Dual kinase-bromodomain inhibitors for rationally designed polypharmacology. *Nature Chemical Biology* **2014**, *10*, 305–312.

(34) Filippakopoulos, P.; Qi, J.; Picaud, S.; Shen, Y.; Smith, W. B.; Fedorov, O.; Morse, E. M.; Keates, T.; Hickman, T. T.; Felletar, I.; Philpott, M.; Munro, S.; McKeown, M. R.; Wang, Y.; Christie, A. L.; West, N.; Cameron, M. J.; Schwartz, B.; Heightman, T. D.; La Thangue, N.; French, C. A.; Wiest, O.; Kung, A. L.; Knapp, S.; Bradner, J. E. Selective inhibition of BET bromodomains. *Nature* **2010**, *468*, 1067–1073.

(35) Picaud, S.; Wells, C.; Felletar, I.; Brotherton, D.; Martin, S.; Savitsky, P.; Diez-Dacal, B.; Philpott, M.; Bountra, C.; Lingard, H.; Fedorov, O.; Müller, S.; Brennan, P. E.; Knapp, S.; Filippakopoulos, P. RVX-208, an inhibitor of BET transcriptional regulators with selectivity for the second bromodomain. *Proceedings of the National Academy of Sciences* **2013**, *110*, 19754–19759.

(36) Hewings, D. S.; Fedorov, O.; Filippakopoulos, P.; Martin, S.; Picaud, S.; Tumber, A.; Wells, C.; Olcina, M. M.; Freeman, K.; Gill, A.; Ritchie, A. J.; Sheppard, D. W.; Russell, A. J.; Hammond, E. M.; Knapp, S.; Brennan, P. E.; Conway, S. J. Optimization of 3,5-Dimethylisoxazole Derivatives as Potent Bromodomain Ligands. *Journal of Medicinal Chemistry* **2013**, *56*, 3217–3227.

(37) Filippakopoulos, P.; Picaud, S.; Fedorov, O.; Keller, M.; Wrobel, M.; Morgenstern, O.; Bracher, F.; Knapp, S. Benzodiazepines and benzotriazepines as protein interaction inhibitors targeting bromodomains of the BET family. *Bioorganic & Medicinal Chemistry* **2012**, *20*, 1878–1886.

(38) Fish, P. V.; Filippakopoulos, P.; Bish, G.; Brennan, P. E.; Bunnage, M. E.; Cook, A. S.; Federov, O.; Gerstenberger, B. S.; Jones, H.; Knapp, S.; Marsden, B.; Nocka, K.; Owen, D. R.; Philpott, M.; Picaud, S.; Primiano, M. J.; Ralph, M. J.; Sciammetta, N.; Trzupek, J. D. Identification of a Chemical Probe for Bromo and Extra C-Terminal Bromodomain Inhibition through Optimization of a Fragment-Derived Hit. *Journal of Medicinal Chemistry* **2012**, *55*, 9831–9837.

(39) Vidler, L. R.; Filippakopoulos, P.; Fedorov, O.; Picaud, S.; Martin, S.; Tomsett, M.; Woodward, H.; Brown, N.; Knapp, S.; Hoelder, S. Discovery of Novel Small-Molecule Inhibitors of BRD4 Using Structure-Based Virtual Screening. *Journal of Medicinal Chemistry* **2013**, *56*, 8073–8088.

(40) Brough, P. A.; Barril, X.; Borgognoni, J.; Chene, P.; Davies, N. G. M.; Davis, B.; Drysdale, M. J.; Dymock, B.; Eccles, S. A.; Garcia-Echeverria, C.; Fromont, C.; Hayes, A.; Hubbard, R. E.; Jordan, A. M.; Jensen, M. R.; Massey, A.; Merrett, A.; Padfield, A.; Parsons, R.; Radimerski, T.; Raynaud, F. I.; Robertson, A.; Roughley, S. D.; Schoepfer, J.; Simmonite, H.; Sharp, S. Y.; Surgenor, A.; Valenti, M.; Walls, S.; Webb, P.; Wood, M.; Workman, P.; Wright, L. Combining Hit Identification Strategies: Fragment-Based and in Silico Approaches to Orally Active 2-Aminothieno[2,3-d] pyrimidine Inhibitors of the Hsp90 Molecular Chaperone. *Journal of Medicinal Chemistry* **2009**, *52*, 4794–4809.
