# Supplementary material for: Absolute Binding Free Energies with OneOPES
Source: J Phys Chem Lett. 2024 Sep 20;15(39):9871–80. doi: 10.1021/acs.jpclett.4c02352 (PMC11457222; doi:10.1021/acs.jpclett.4c02352)
Supplement: Supplementary file 2 — jz4c02352_si_002.pdf [file jz4c02352_si_002.pdf]

jz-2024-023524.R1

Name: Peer Review Information for "Absolute Binding Free Energies with OneOPES"

First Round of Reviewer Comments

Reviewer: 1

Comments to the Author

Karrenbrock et al. presents the application a a recent new method for calculating absolute binding free energies termed OneOPES (what it stands for is not explained or I may have overlooked it. At least it is not explained when first mentioning the approach in the Introduction). The method is based on metadynamics and replica exchange simulations along 2 main CVs combined with additional CVs to improve sampling and accelrate convergence. THe application 2 two test systems indicates promising results. I think the method is a nice additional tool for calculation of absolute binding free energies. I have, however, a few comments:

1. The authors use the method of spatial separation of ligand and protein for absolute binding free energy calculation instead of the alchemical approach. One argument is that in the latter approach one may not sample conformational changes in the protein necessary for binding or unbinding. However, for spatial separation along one distance type CV one major problem is possible sterical overlap between ligand and receptor (not occuring in case of using the alchemical pathway). To avoid this overlap the receptor has often to undergo large conformational changes difficult to sample. When looking at the additional CVs used by the authors I cannot see a CV that specifically deals with this problem. Hence, is the approach only usefiul for ligands binding at the protein surface and not at some burried sites? This should be discussed.

2. Especially for the first test system the magnitude of binding of all ligands is reproduced overall quite well. However, the ordering of binding affinity of the ligands is quite different from experiment. Especially, this ordering can be important for drug design efforts. The authors should discuss this issue.

3. The authors indicate that in the order of 700 ns sampling time per replica (hence several us when using 8 or so replicas) is necessary to achieve convergence, I remember that in the method used by Woo and Roux (PNAS, 2005) with several geometric restraints much shorter simulations (< 100 ns total time) where sufficient to obtain good convergence and good agreement with experiment. Furthermore, alchemical absolute binding free energy calculations (if the ligand is not too large) can be done with significantly shorter simulations (see for example a recent paper by Gilson and coworkers in JCTC). The authors should explain and discuss why the new method seems to be computationally more (not less) demanding than previous methods.

4. There is little comparison of the method with already available methods for absolute binding free energy calculations. There are a number of automatic methods/protocols by Fu et al. and by the Gilson to setup and run such simulations achieving also high accuracy and efficiency. Although cited a comparison is not presented.

Reviewer: 2

Comments to the Author

The paper by the Gervasio group represents a significant contribution for the key problem of calculating absolute binding free energies in ligand-protein interaction studies.

The study is very accurate, well presented and well written. The conclusions are consistent with hypotheses and the theoretical part is solidly implemented.

The work done on two systems adds value to the significance of the paper.

A small potential modification to the paper, may be extending the discussion to the possibility of applying their approach to the sampling of cryptic or allosteric pockets, which may be an interesting development as in the case of Hsp90.

Author's Response to Peer Review Comments:

Dear The Journal of Physical Chemistry Letters Editor,

We thank the reviewers for their constructive comments and questions. We have revised and improved the manuscript by adding some further discussions and citations. We have also changed the sign of all binding free energies in accordance with standard convention.

Below, we report the reviewers' comments in black and our response in blue for each question or comment. We also highlighted in blue the changes that we made in the revised version of the manuscript.

Kind regards,

Francesco Luigi Gervasio

Reviewer: 1

Karrenbrock et al. presents the application a a recent new method for calculating absolute binding free energies termed OneOPES (what it stands for is not explained or I may have overlooked it. At least it is not explained when first mentioning the approach in the Introduction). The method is based on metadynamics and replica exchange simulations along 2 main CVs combined with additional CVs to improve sampling and accelrate convergence. The application to two test systems indicates promising results. I think the method is a nice additional tool for calculation of absolute binding free energies.

Regarding the meaning of the acronym OneOPES, it stands for One On-the-fly Probability Enhanced Sampling, a multi-replica implementation of the OPES method, which in turn is an evolution of Well Tempered MetaDynamics (WT-MetaD). We have now defined the acronym in the manuscript.

I have, however, a few comments:

1. The authors use the method of spatial separation of ligand and protein for absolute binding free energy calculation instead of the alchemical approach. One argument is that in the latter approach one may not sample conformational changes in the protein necessary for binding or unbinding. However, for spatial separation along one distance type CV one major problem is possible sterical overlap between ligand and receptor (not occurring in case of using the alchemical pathway). To avoid this overlap the receptor has often to undergo large conformational changes difficult to sample. When looking at the additional CVs used by the authors I cannot see a CV that specifically deals with this problem. Hence, is the approach only useful for ligands binding at the protein surface and not at some buried sites? This should be discussed.

While the systems presented in the manuscript represent accessible binding sites, the approach

is not inherently limited to capture only such cases. The OneOPES strategy is very flexible and can be adapted to handle conformational changes in the receptor by adding appropriate auxiliary CVs. Indeed, while we focus here on systems in which flexible ligands of different sizes bind to relatively rigid pockets, we plan to test the approach on systems with deeply buried sites requiring conformational changes or even cryptic pockets in a follow-up article (see also our response to Reviewer 2).

2. Especially for the first test system the magnitude of binding of all ligands is reproduced overall quite well. However, the ordering of binding affinity of the ligands is quite different from experiment. Especially, this ordering can be important for drug design efforts. The authors should discuss this issue.

It is indeed challenging to obtain the correct ranking for compounds that are close in binding free energies and differ by less than 1 kcal mol^{-1} as the accuracy of the protein, water and especially ligand force fields is typically worse than this. Kendall's τ is routinely used to measure the quality of the ranking and our results ($\tau = 0.53 \pm 0.08$ and $\tau = 0.59 \pm 0.29$ for the BRD4 and Hsp90 systems, respectively) indicate that we are in line with previously reported results on analogous systems (Gapsys et al. Commun. Chem 2021). In particular, τ values above 0.5 indicate a strong monotonic relationship where the ranks of the variables are globally aligned. We would like to emphasise that in the context of drug design it is crucial to discriminate between affinities that differ by more than 1 kcal mol^{-1} in experiments, and in this respect our results reflect well the experimental ordering.

3. The authors indicate that in the order of 700 ns sampling time per replica (hence several us when using 8 or so replicas) is necessary to achieve convergence, I remember that in the method used by Woo and Roux (PNAS, 2005) with several geometric restraints much shorter simulations (¡ 100 ns total time) where sufficient to obtain good convergence and good agreement with experiment. Furthermore, alchemical absolute binding free energy calculations (if the ligand is not too large) can be done with significantly shorter simulations (see for example a recent paper by Gilson and coworkers in JCTC). The authors should explain and discuss why the new method seems to be computationally more (not less) demanding than previous methods.

While other methods (including some alchemical approaches) are reported to achieve convergence in shorter simulation times, they typically achieve so by focusing the sampling on a reduced number of conformations and by strongly optimizing the strategy on the systems under study. This optimization tends to require substantial preliminary information.

For CV-based calculations, the choice of optimal CVs is crucial, but optimal CVs are system-dependent and generally unknown. For this reason, it has been repeatedly shown that free energy convergence with general (and sub-optimal) CVs typically requires longer sampling times and multiple replicas (Evans et al. JCTC 2020). As for alchemical calculations, the knowledge of the experimental binding pose, and preferred ligand conformations are typically leveraged to speed up convergence (with e.g. Boresh-like restraints). When the cystallographic binding pose is not known much longer sampling times are needed, as shown for example in our recent paper (Karrenbrock et al. JPCB 2024).

Regarding the work of Woo and Roux from 2005, it is indeed one of the cornerstones of CV-based binding free energies similarly to the first use of Metadynamics by Gervasio et al. from the same year. However, we now know much more about the convergence of CV-based algorithms and as the reviewer correctly pointed out, early work resorted to system specific knowledge and (in the case of Umbrella Sampling) to several restraints to extract results in a shorter simulation time, sacrificing generality. Furthermore, Umbrella Sampling is known to be very dependent on the choice of CVs, umbrella windows and especially the starting configuration used in each umbrella window (Lichtinger and Biggin, JCTC 2023). Achieving a solid convergence on a number of systems with a simple 1-D umbrella sampling along a simple distance CV is extremely challenging, so a direct comparison between the two would be unhelpful in this context.

Our approach, on the other hand, aims to produce converged absolute binding free energies in a more general manner, by using rather generic CVs and not targeting sampling on a subset of previously known states. We believe that such an agnostic approach, although generally requiring longer but affordable simulations, can better inform drug discovery campaigns where high quality structural information is typically limited.

From a practical point of view, a key aspect is that the sampling is not serial and replicas are run in parallel. Modern computing platforms that include GPUs are ideal for parallel computing and, in this respect, our strategy is reasonably affordable.

4. There is little comparison of the method with already available methods for absolute binding free energy calculations. There are a number of automatic methods/protocols by Fu et al. and by the Gilson to setup and run such simulations achieving also high accuracy and efficiency. Although cited a comparison is not presented.

The manuscript is mainly focused in describing the key aspects of OneOPES for ligand binding and highlight its use on two standard protein-ligand set of systems. At this stage, the most relevant comparison of our method because of method similarity is with Funnel MetaDynamics and in fact we presented such comparison in Supplementary Table 6 and Supplementary Figure 8 for BRD4.

Reviewer: 2

The paper by the Gervasio group represents a significant contribution for the key problem of calculating absolute binding free energies in ligand-protein interaction studies. The study is very accurate, well presented and well written. The conclusions are consistent with hypotheses and the theoretical part is solidly implemented. The work done on two systems adds value to the significance of the paper. A small potential modification to the paper, may be extending the discussion to the possibility of applying their approach to the sampling of cryptic or allosteric pockets, which may be an interesting development as in the case of Hsp90.

We have extended our conclusions to include potential future applications, particularly in the context of sampling cryptic pockets. For instance, applying our strategy to systems such as those discussed in Borsatto et al. JCTC 2024 would be highly valuable. In that study,

we used SWISH-X, a method that we developed for sampling the opening (and closing) of cryptic pockets. By applying OneOPES to those systems and combining it with CVs specifically designed to sample the dynamics of cryptic pocket opening and closing, we could quantitatively capture the free energy differences between open and closed states. This approach would also allow us to perform ligand binding calculations that simultaneously account for pocket conformational changes and ligand binding, thereby providing a more comprehensive understanding of the binding mechanism.
